# Immunogenicity and efficacy of fourth BNT162b2 and mRNA1273 COVID-19 vaccine doses; three months follow-up

**DOI:** 10.1038/s41467-022-35480-2

**Published:** 2022-12-13

**Authors:** Michal Canetti, Noam Barda, Mayan Gilboa, Victoria Indenbaum, Michal Mandelboim, Tal Gonen, Keren Asraf, Yael Weiss-Ottolenghi, Sharon Amit, Ram Doolman, Ella Mendelson, Dror Harats, Laurence S. Freedman, Yitshak Kreiss, Yaniv Lustig, Gili Regev-Yochay

**Affiliations:** 1 The Infection Prevention & Control Unit, Sheba Medical Center, Tel Hashomer, Ramat Gan, Israel; 2 Sackler School of Medicine, Tel-Aviv University, Tel Aviv, Israel; 3 ARC Innovation Center, Sheba Medical Center, Tel Hashomer, Ramat Gan, Israel; 4 Software and Information Systems Engineering, Ben-Gurion University of the Negev, Be’er Sheva, Israel; 5 Department of Biomedical Informatics, Harvard Medical School, Boston, MA USA; 6 Central Virology Laboratory, Public Health Services, Ministry of Health, Tel-Hashomer, Ramat Gan, Israel; 7 The Dworman Automated-Mega Laboratory, Sheba Medical Center, Tel-Hashomer, Ramat-Gan, Israel; 8 Clinical Microbiology, Sheba Medical Center, Tel Hashomer, Ramat Gan, Israel; 9 General Management, Sheba Medical Center, Tel Hashomer, Ramat Gan, Israel; 10 Biostatistics and Biomathematics Unit, Gertner Institute of Epidemiology and Health Policy Research, Sheba Medical Center, Tel Hashomer, Israel; 11 Vaccine Research Center, National Institute of Allergy and Infectious Diseases, National Institutes of Health, Bethesda, MD USA

**Keywords:** Clinical trials, SARS-CoV-2, RNA vaccines, Viral infection

## Abstract

Booster doses for the ongoing COVID-19 pandemic are under consideration in many countries. We report a three-month follow-up of 700 participants in a fourth vaccine dose study, comparing BNT162b2 and mRNA1273, administered four months after a third BNT162b2 dose. The primary outcomes are the levels of IgG, neutralizing antibodies, and microneutralization and the secondary outcomes are the levels of IgA and T cell activation, and clinical outcomes of SARS-CoV-2 infection and substantial symptomatic disease. Waning of the immune response is evident during follow-up, with an 11% (β = 0.89, 95% CI, 0.88–0.9) and 21% (β = 0.79, 95% CI, 0.76–0.82) multiplicative decay per week of IgG and neutralizing antibodies, respectively, in the mRNA1273 group, and of 14% (β = 0.86, 95% CI, 0.86–0.87) and 26% (β = 0.74, 95% CI, 0.72–0.76), respectively, in the BNT162b2 group. Direct neutralization of Omicron variants is low relative to ancestral strains. Cumulatively over the study period, both vaccines show little efficacy against infection but were highly efficacious against substantial symptomatic disease [89% [(IRR 0.11, 95% CI, 0.02–0.37) and 71% (IRR 0.29, 95% CI, 0.13–0.57) for mRNA1273 and BNT162b2, respectively]. These results are informative for further boosting policy-making. Trial registration numbers (clinicaltrials.gov): NCT05231005 and NCT05230953.

## Abstract (web summary)

Here the authors provide immunogenicity and efficacy data at 3-month follow-up for vaccinees who have received a fourth dose of either mRNA1273 or BNT162b2. Both vaccines were highly effective against substantial symptomatic disease, but had little effect against SARS-CoV-2 infection.

## Introduction

Since its emergence in December 2019, Coronavirus disease 2019 (COVID-19) has claimed the lives of more than 6 million people^1^. During this period, multiple measures have been taken to reduce infection, disease, and death rates, including the messenger RNA (mRNA) COVID-19 vaccines, Pfizer-BioNTech (BNT162b2), and Moderna (mRNA1273)^2,3^.

Following the receipt of two mRNA vaccine doses, a significant humoral immune response was reported^4^, as well as a decrease in the rates of infection, infectivity, disease, hospitalization, and death^5–8^. However, the waning of this protective effect was evident after a few months, as observed by decreases in immunogenicity and vaccine effectiveness against infection and disease^9–11^. This decline, along with the emergence of novel variants of concern (VOC), led to the rollout of a third dose. Initially, receipt of the third dose was reported to decrease infection and severe illness rates and to generate a stronger immune response in comparison to that induced by the first and second doses^12–14^. Yet, shortly after, vaccine effectiveness was reported to wane^15–17^, probably due to the emergence of the highly transmissible Omicron VOC^18–20^, as well as the waning of the immune response^21^. This raised the question of whether a fourth vaccine dose was needed.

On December 27, 2021, we began an open-label, nonrandomized, clinical study, assessing the safety, immunogenicity, and efficacy of the fourth-dose vaccines, either 50 μg of mRNA1273 or 30 μg of BNT162b2, in health care workers (HCW) who previously received three doses of BNT162b2. The study was carried out during the time when Omicron was the predominant VOC in Israel. We previously reported the short-term (up to one month) results of this study, showing that the fourth vaccine dose was safe and led to an immune response comparable to that of the third dose, but resulted in a vaccine efficacy (VE) of only 11–30%^22^, which was lower than that achieved after the previous doses^23,24^.

The durability of the immune response and the longer-term vaccine efficacy of the fourth dose have not yet been studied. Here we report interim results from a 102-day follow-up of the fourth dose study (the overall follow-up was 180 days), examining immune response and clinical outcomes, and comparing the BNT162b2 and mRNA1273 mRNA COVID-19 vaccines.

## Results

This open-label controlled intervention study was conducted at the Sheba Medical Centre (SMC), the largest tertiary hospital in Israel. Eligible participants were HCW who had previously received three doses of the BNT162b2 vaccine, had no known history of severe acute respiratory syndrome coronavirus 2 (SARS-CoV-2) infection, and had recorded baseline IgG levels <700 BAU (full eligibility criteria are described in the Methods section). Participants (*n* = 700) were enrolled to either receive the BNT162b2 vaccine, the mRNA1273 vaccine, or to serve as controls for one or both of the two arms. Participants were followed up for immune response and clinical outcomes.

**Fig. 1:**
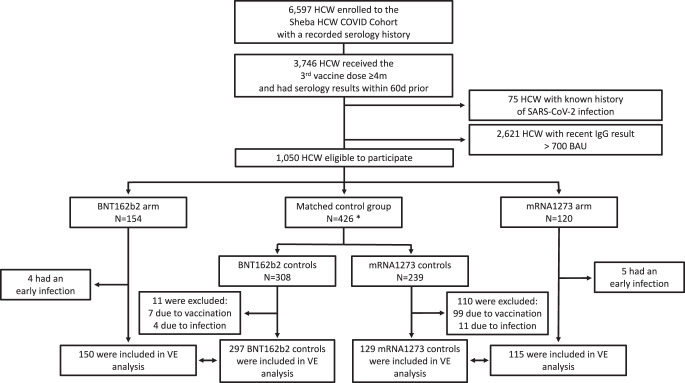
Study population flow chart. A chart detailing the number of individuals included and excluded according to each eligibility criteria in the study. Participants (vaccine recipients and controls) who experienced early infection (positive SARS-CoV-2 test before day 8) were excluded from the vaccine efficacy analysis. Controls who received the fourth dose vaccine dose before day 8 were also excluded. HCW, Health care workers, VE, Vaccine efficacy. *Participants in the control group were allowed to serve as controls of both intervention arms.

Of a total of 6597 HCW enrolled to the Sheba HCW COVID Cohort^4,9,25,26^, 1050 were eligible to participate. Of these, 154 were enrolled to the BNT162b2 arm on December 27–28, 2021. One week later, on January 5–6, 2022, 120 participants were enrolled to the mRNA1273 arm. From the remaining eligible 776 participants, a total of 426 controls were age-matched in a 2:1 ratio to participants in each arm (121 served as controls for both intervention arms). Participants who experienced early infection (until day 8) were excluded. Additionally, controls who received the fourth vaccine dose (outside the trial) within the first eight days were excluded on the day of vaccination. Initially, more controls of the mRNA1273 group received the vaccine, which became available to the HCW population on January 2, 2022, due to the one-week difference in enrollment between the vaccine groups. See Fig. 1 for the complete study population flow chart.

The baseline characteristics of the study population are described in Table 1. Baseline characteristics of the population used in the analysis of the clinical outcomes (which excludes those infected during the first 7 days of follow-up) are described in Table S1.

**Table 1:** Baseline characteristics of the study population

		BNT162b2	Control (BNT162b2)	mRNA1273	Control (mRNA1273)
Participants enrolled	*N*	154	308	120	239
Female sex	*N* (%)	91 (59)	218 (71)	81 (67)	178 (74)
Male sex	*N* (%)	63 (41)	90 (29)	39 (33)	61 (26)
Age	Median (range)	60.85 (30.44–85.31)	61.33 (30.27–89.61)	55.97 (29.26–86.94)	56.85 (29.21–89.61)
BMI (kg/m^2^)	Median (IQR)	26.03 (23.11–28.4)	25.48 (23.15–28.12)	24.77 (22.4–28.23)	25.48 (22.96–28.76)
Missing data	%	16		14	
**Comorbidities**					
0	*N* (%)	92 (60)	195 (63)	80 (67)	150 (63)
1	*N* (%)	41 (27)	51 (17)	28 (23)	41 (17)
≥2	*N* (%)	21 (13)	62 (20)	12 (10)	48 (20)
Missing data	%	9	9		
Hypertension	*N* (%)	42 (27)	32 (12)	23 (19)	22 (11)
Diabetes	*N* (%)	14 (9)	18 (7)	13 (11)	14 (7)
Lung disease	*N* (%)	7 (5)	8 (3)	7 (6)	4 (2)
Heart disease	*N* (%)	11 (7)	7 (3)	7 (6)	6 (3)
Liver disease	*N* (%)	1 (0.6)	2 (1)	1 (1)	1 (0.5)
Chronic kidney disease	*N* (%)	1 (0.6)	2 (1)	3 (3)	1 (0.5)
Autoimmune disease	*N* (%)	12 (8)	20 (8)	4 (3)	15 (7)
Immunosuppression	*N* (%)	3 (2)	4 (2)	2 (2)	1 (0.5)
Missing data	%	9		10	
**Profession**					
Physician	*N* (%)	41 (27)	50 (16)	35 (29)	36 (15)
Nurse	*N* (%)	34 (22)	77 (25)	21 (18)	70 (29)
Allied health professions	*N* (%)	34 (22)	59 (19)	35 (29)	36 (15)
Administration or maintenance	*N* (%)	43 (28)	122 (40)	26 (22)	97 (41)
Other personnel	*N* (%)	2 (1)	0	3 (2)	0

*BMI* body mass index.

### Immunogenicity

**Fig. 2:**
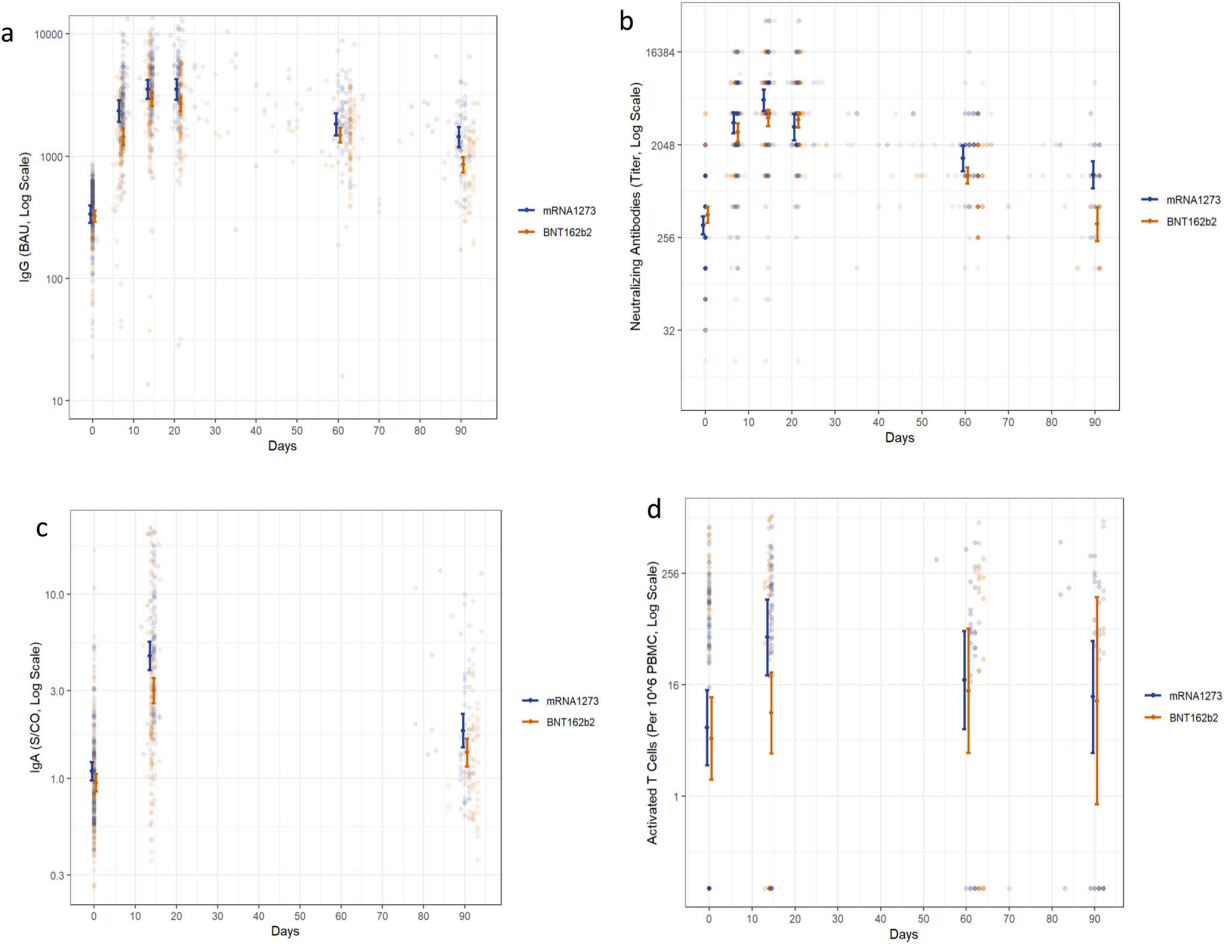
Immunogenicity after the fourth dose messenger RNA (mRNA) vaccines. A three-month follow-up following mRNA1273 or BNT162b2 fourth dose vaccines. **a** IgG antibodies (for mRNA1273: *n* = 117, *n* = 107, *n* = 98, *n* = 74, *n* = 70, *n* = 56, for BNT162b2: *n* = 149, *n* = 142, *n* = 136, *n* = 129, *n* = 92, *n* = 68, biologically independent samples, on days 0, 7, 14, 21, 60, and 90, respectively), **b** neutralizing antibodies (for mRNA1273: *n* = 116, *n* = 107, *n* = 78, *n* = 74, *n* = 69, *n* = 33, for BNT162b2: *n* = 149, *n* = 142, *n* = 136, *n* = 128, *n* = 93, *n* = 25, biologically independent samples, on days 0, 7, 14, 21, 60, and 90, respectively), **c** IgA antibodies (for mRNA1273: *n* = 114, *n* = 97, *n* = 56, for BNT162b2: *n* = 149, *n* = 134, *n* = 68, biologically independent samples, on days 0, 14, and 90, respectively), and **d** number of activated T cells (for mRNA1273: *n* = 54, *n* = 35, *n* = 26, *n* = 27, for BNT162b2: *n* = 56, *n* = 52, *n* = 24, *n* = 11, biologically independent samples, on days 0, 14, 60, and 90, respectively). In each figure, the raw values are presented on a log Y scale. Geometric mean titers and their 95% confidence intervals at each planned encounter are overlaid on the plot. Source data are provided as a Source Data file.

Crude values of the serological markers are presented in Table S2 and Fig. 2. The different immunological markers show a similar trend of reaching a maximum level 2–3 weeks following vaccination and then slowly declining. During the first month after vaccination, a 9–10-fold increase was demonstrated in anti-RBD immunoglobulin G (IgG) titer in both vaccine recipient groups. The waning of this response was observed over time, with IgG geometric mean titer (GMT) of 1442 binding antibody units (BAU) (95% CI, 1194–1741) in the mRNA1273 group and 854 BAU (95% CI, 738–989) in the BNT162b2 group observed after 90 days. In neutralizing antibodies, the increase to peak levels was of 16.5-fold in the mRNA1273 group and ninefold in the BNT162b2 group. After 90 days, the GMT of neutralizing antibodies in the mRNA1273 group was 1046 (95% CI, 772–1417), while the BNT162b2 group had a neutralizing antibody titer of 347 (95% CI, 238–507), close to their pre-fourth dose titers. Fourteen days following the fourth dose, a 4.5-fold increase in the mRNA1273 group and a threefold increase in the BNT162b2 group was observed in anti-RBD immunoglobulin A (IgA), reaching GMT of 4.63 sample-to-cutoff ratio (s/co) (95% CI, 3.88–5.53) and 3 s/co (95% CI, 2.57–3.53), respectively. After three months, IgA levels declined to 1.82 (95% CI, 1.48–2.25) and 1.39 (95% CI, 1.16–1.65), respectively. The number of activated T cells elevated in the mRNA1273 group from 6 per 10^6^ peripheral blood mononuclear cells (PBMC) (95% CI, 2–14) to 52 per 10^6^ PBMC (95% CI, 20–134) and declined to 11 per 10^6^ PBMC (95% CI, 3–48) after three months, while smaller changes were observed in the BNT162b2 group.

Using an adjusted model for each vaccine, we estimated in the mRNA1273 vaccine arm a 11% [geometric mean ratio (GMR) = 0.89, 95% CI, 0.88–0.9] multiplicative decay per week in IgG, 21% (GMR = 0.79, 95% CI, 0.76–0.82) in neutralizing antibody titers, and 10% (GMR = 0.9, 95% CI, 0.88–0.92) in IgA. In the BNT162b2 vaccine arm, we estimated a multiplicative decay per week of 14% (GMR = 0.86, 95% CI, 0.86–0.87), 26% (GMR = 0.74, 95% CI, 0.72–0.76), and 8% (GMR = 0.92, 95% CI, 0.9–0.93), respectively. No significant decay was shown in the number of activated T cells in both groups [10% (GMR = 0.9, 95% CI, 0.77–1.05) and 10% (GMR = 0.9, 95% CI, 0.74–1.09), respectively] (Table S3a).

Using an adjusted model comparing the two mRNA vaccines, we demonstrated a significant difference in the decline of IgG and neutralizing antibody titers between the two vaccines, with a further multiplicative decay of 2% per week (GMR = 0.98, 95% CI, 0.96–0.99) and 7% (GMR = 0.93, 95% CI, 0.89–0.98), respectively, in the BNT162b2 group. Furthermore, we showed a 29% (GMR = 0.71, 95% CI, 0.57–0.89) lower IgA peak in the BNT162b2 group (Table S3b).

**Fig. 3:**
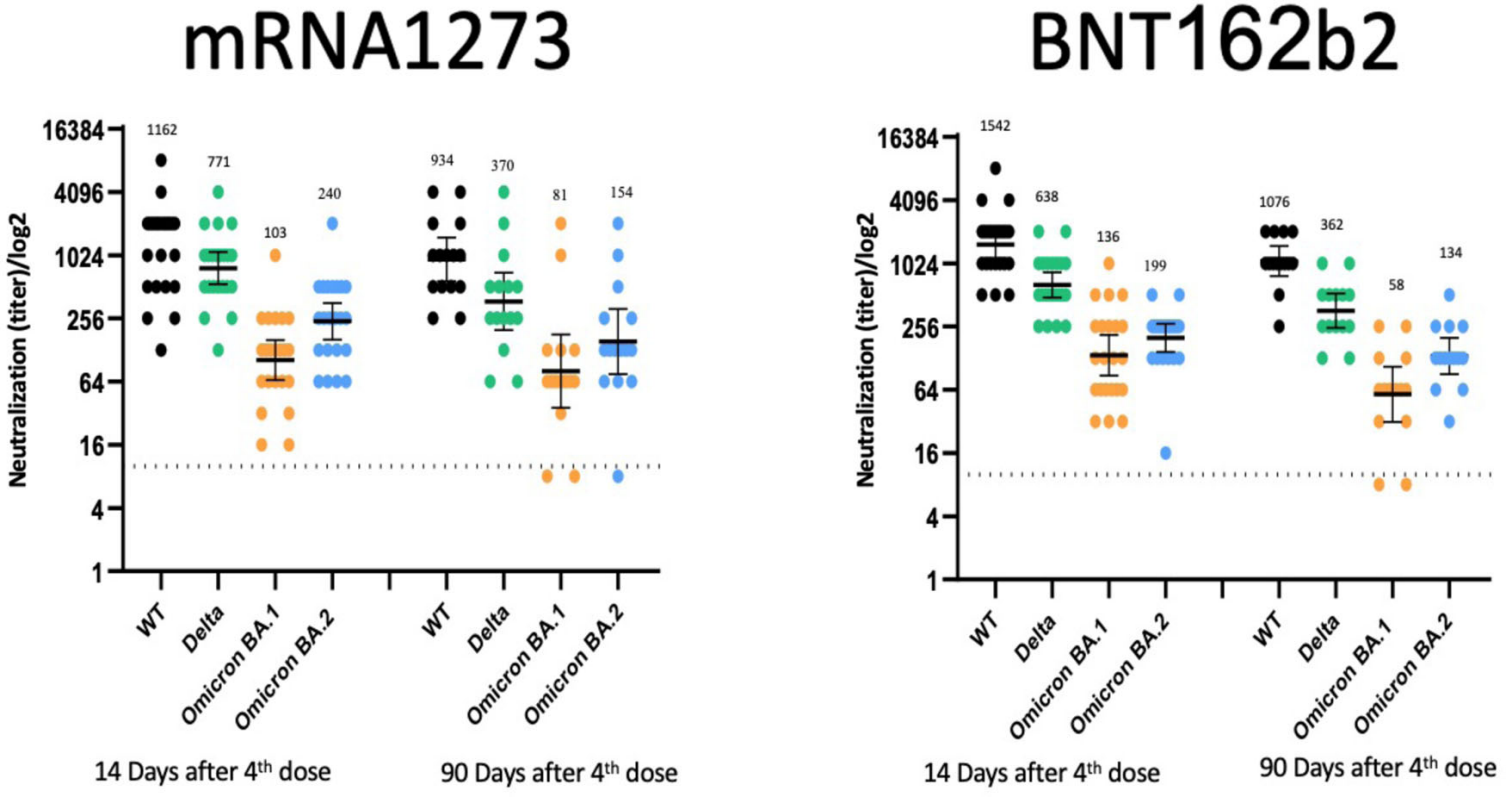
Direct neutralization titers against different strains. Live virus neutralization efficiency follow-up at 14- and 90-days post-vaccination with mRNA1273 or BNT162b2 fourth dose vaccines against different strains [Index virus, Delta VOC, Omicron BA.1 VOC, and Omicron BA.2 VOC] (for mRNA1273: *n* = 21 and *n* = 14, for BNT162b2: *n* = 20 and *n* = 14 after 14 and 90 days, respectively). Raw values are presented on a log Y scale, with geometric mean titers and their 95% confidence intervals overlaid. Source data are provided as a Source Data file.

Higher direct neutralization titers were observed for the wild-type strain and the Delta VOC than for the Omicron VOC. All direct neutralization titers demonstrated waning between days 14 and 90 post-vaccination. In addition, direct neutralization results for the Omicron BA.2 VOC were comparable and perhaps higher than for Omicron BA.1 (Fig. 3).

### Vaccine efficacy

**Fig. 4:**
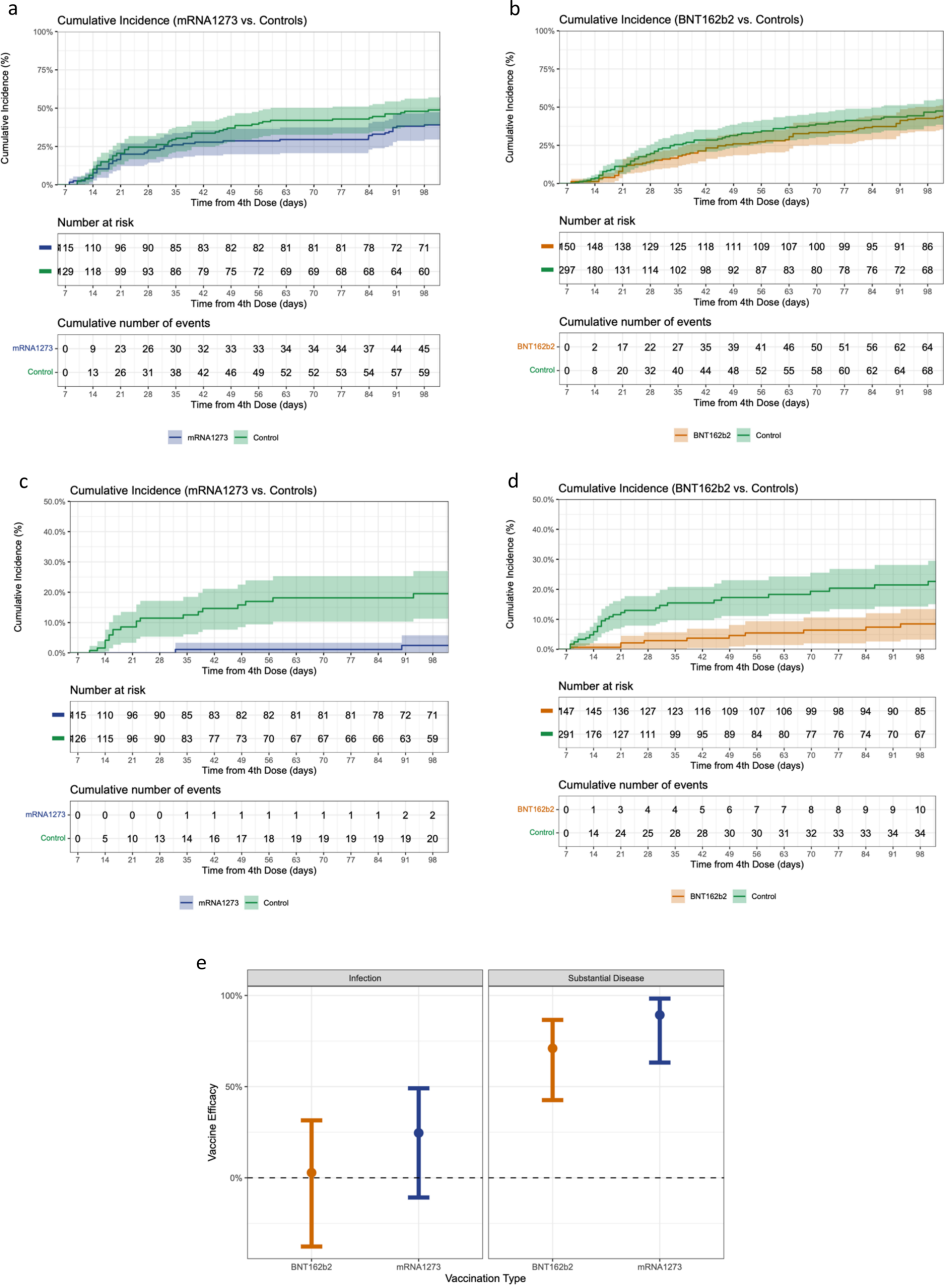
Cumulative incidence and vaccine efficacy against infection and substantial symptomatic disease. Cumulative incidence of **a** all SARS-CoV-2 infections among the mRNA1273 fourth dose arm and their matched controls, **b** all SARS-CoV-2 infections among the BNT162b2 fourth dose arm and their matched controls, **c** substantial symptomatic disease among the mRNA1273 fourth dose arm and their matched controls, and **d** substantial symptomatic disease among the BNT162b2 fourth dose arm and their matched controls. Vaccine efficacy against infection (for mRNA1273: *n* = 115, for BNT162b2: *n* = 150) and substantial symptomatic disease (for mRNA1273: *n* = 115, for BNT162b2: *n* = 147) for both vaccines compared to receiving three vaccine doses, estimated using an adjusted Poisson regression, is demonstrated in plot (**e**). In each figure, the outcome is presented using a 95% confidence interval. Source data are provided as a Source Data file.

Cumulative incidence was estimated in both arms for two clinical outcomes: (i) infection, defined by positive SARS-CoV-2 test, with or without symptoms, as determined by active surveillance, and (ii) substantial symptomatic disease, as defined by two or more days in which the participant was mostly in bed due to feeling unwell (Table S4 and Fig. 4). During follow-up, none of the participants experienced severe outcomes (e.g., severe disease, hospitalization, or death).

Within 102 days of follow-up, 40–50% of the study population were infected, with only a little difference between the intervention arms and their controls. In contrast, the cumulative incidence of substantial symptomatic disease was higher in the control groups: 2o% (incidence = 0.2, 95% CI, 0.11–0.27) in the controls compared with 3% (incidence = 0.03, 95% CI, 0–0.06) in the mRNA1273 vaccine arm, and 23% (incidence = 0.23, 95% CI, 0.15–0.3) and 9% (incidence = 0.09, 95% CI, 0.03–0.13) in the controls and vaccine recipients in the BNT162b2 arm, respectively.

A multivariable model, adjusted for age and sex, estimated a VE against SARS-CoV-2 infection of 25% (IRR 0.75, 95% CI, 0.51–1.11) for mRNA1273 and 3% (IRR 0.97, 95% CI, 0.69–1.38) for BNT162b2, when compared to receiving three vaccine doses. The VE against the substantial symptomatic disease was 89% (IRR 0.11, 95% CI, 0.02–0.37) in the mRNA1273 group and 71% (IRR 0.29, 95% CI, 0.13–0.57) in the BNT162b2 group (Table 2, Table S5, and Fig. 4e).

**Table 2:** COVID-19 Vaccine efficacy for infection and substantial symptomatic disease

A. COVID-19 VE for infection					
Variable	Person-days	Positive cases	Incidence rate per 1000 person-days	Crude VE	Adjusted VE
mRNA1273 controls (*N* = 129)	7577	61	8.05	-ref-	-ref-
mRNA1273 (*N* = 115)	8029	45	5.6	30.4%	24.6% (−10.8–49.1%)
BNT162b2 controls (*N* = 297)	9669	69	7.14	-ref-	-ref-
BNT162b2 (*N* = 150)	10,697	66	6.17	13.6%	2.8% (−37.7–31.5%)

B. COVID-19 VE for substantial symptomatic disease					
mRNA1273 controls (*N* = 126)	7377	20	2.71	-ref-	-ref-
mRNA1273 (*N* = 115)	8029	2	0.25	90.8%	89.3% (63.2–98.3%)
BNT162b2 controls (*N* = 291)	9395	35	3.73	-ref-	-ref-
BNT162b2 (*N* = 147)	10,541	10	0.95	74.5%	71% (42.63–86.63%)

Vaccine efficacy is calculated as 1-IRR.

## Discussion

We conducted an interventional study, comparing the immune response and the vaccine efficacy of the fourth dose of mRNA1273 and BNT162b2 vaccines with a 102-day follow-up. We report waning of the immune response across a wide range of immunologic markers—IgG, IgA, neutralizing antibodies, and direct neutralization- similar to that described after the third vaccine dose^22^. A similar change was not observed in the number of activated T cells. Furthermore, we found that the peak immune response was lower and the waning faster in the BNT162b2 group. Despite this finding, VE for preventing substantial symptomatic disease from the Omicron VOC was high in both groups compared to receiving three vaccine doses [89% (IRR 0.11, 95% CI, 0.02–0.37) and 71% (IRR 0.29, 95% CI, 0.13–0.57), for mRNA1273 and BNT162b2, respectively]. Finally, both vaccines showed little efficacy in preventing infection by the Omicron VOC in our study cohort compared to three vaccine doses [VE of 25% (IRR 0.75, 95% CI, 0.51–1.11) and 3% (IRR 0.97, 95% CI, 0.69–1.38) for mRNA1273 and BNT162b2, respectively].

We report a waning of the immune response following a fourth vaccine dose. After three months, a significant decay of IgG, IgA, and neutralizing antibodies was evident in both vaccines, with a further decay in IgG and neutralizing antibodies in the BNT162b2 group. We were able to show that after three months, the immunological markers tested lost much of the elevation gained following the receipt of the fourth dose and that some nearly returned to their baseline point as measured four months after the third vaccine dose. A decay of the immune response was previously reported after both the second and third vaccine doses^9,21,27,28^. We previously showed similar waning of IgG and neutralizing antibodies after the third BNT162b2 dose^21^. Therefore, the data presented here further support our previous results^22^ that a fourth vaccine dose temporarily restores antibody levels but does not change antibody dynamics. Immunologically, this suggests that periodic booster doses will be necessary to maintain high antibody levels.

Interestingly, a larger increase in T cell activation was noted following the administration of a fourth mRNA1273 dose but not after a BNT162b2 fourth dose. This difference, however, was not maintained and T cell activity was similar between the two vaccines two and three months following the fourth dose. Future studies should directly investigate the role of cellular response by examining the correlation between activated T cells and protection from SARS-CoV-2 infection or symptomatic COVID-19.

Due to its involvement in mucosal immunity, we also investigated IgA levels. Our results point to similar kinetics for IgA, IgG, and neutralizing antibodies: a rapid induction following the fourth dose followed by a slow decline. A recent study implicated serum IgA levels with protection against SARS-CoV-2 infection^29^, thus suggesting that IgA may also contribute to mRNA vaccines protective effects.

Higher levels of humoral immune response were previously reported for two doses of the mRNA1273 vaccine compared to the BNT162b2 vaccine^30^. Recently, Kaplonek et al. reported a more detailed analysis, showing further immunological benefits of mRNA1273 compared to BNT162b2 vaccine, among these greater inductions of IgA by mRNA1273^31^, which we did not observe. Possible explanations for the small benefit of mRNA1273 in our study could be the different doses of the two vaccines (30 μg vs. 50 μg for BNT162b2 and mRNA1273, respectively), the different formulations^32^, or that a heterogeneous boost may be beneficial in inducing humoral and cellular responses compared to homogeneous boosting, as has been previously suggested^33,34^. Here we demonstrated that a single mRNA1273 vaccine dose, given as a fourth dose after three BNT162b2 doses, achieves slightly higher peak levels and a lower decay rate of different immunologic markers compared with four consecutive doses of BNT162b2.

Our study did not show that the fourth vaccine dose was effective in protecting against infection (compared to receiving three vaccine doses). Indeed, nearly half of the study population were infected with SARS-CoV-2, despite receiving three or four vaccine doses. Yet, we show high VE against substantial symptomatic disease, defined as disease leading to spending at least two days in bed feeling unwell. Significant reductions in hospitalization and death rates after receipt of a fourth BNT162b2 dose were previously reported^35^. Here, we demonstrated high effectiveness of a fourth dose even in non-severe but substantially symptomatic disease over three months of follow-up. This finding is important, as severe disease is now relatively rare^36–38^ in healthy vaccinated individuals, and protection against substantial symptomatic disease is important for maintaining an open economy, especially in light of the high infection rates of Omicron and its sub-lineages.

We were unable to determine SARS-CoV-2 lineage for participants who were infected, but Omicron BA.1 was the predominant strain in Israel during the follow-up period and was likely the infecting VOC in most, if not all, cases. The finding of a low VE against infection during follow-up is further supported by the low titer for Omicron VOC observed in the direct neutralization assay, compared with other variants, thus suggesting that immune evasion might have a significant role in the low VE observed. These data suggest that an Omicron-modified or a new mucosal vaccine may be needed to provide better protection from infection by future booster vaccine doses.

Our study has several limitations. First, treatment allocation was not randomized, and confounding is possible. While we did attempt to account for this by adjusting for age and sex using matching and multivariable regression, other variables not adjusted for could still bias the estimates. Second, follow-up intensity varied over the study period, with weekly COVID-19 tests performed during the first month but at larger intervals as time passed. This could result in misclassification bias. We attempted to mitigate this bias by sending weekly reminders to participants through text messages and emails encouraging them to undergo testing. Third, our study is relatively small, and the confidence intervals are correspondingly wide for the vaccine efficacy outcomes. Fourth, the study included only participants with IgG levels below 700, which can affect the generalizability of the results. Finally, individuals infected during the study period were eligible to receive treatment with anti-viral drugs (e.g., Paxlovid) if their risk for the severe disease was high. While this could bias the VE outcomes, the relatively young and healthy profile of the HCW in our sample resulted in few patients receiving such treatment.

To conclude, our data provide evidence regarding the waning of the immune response during the three months following receipt of the fourth mRNA COVID-19 vaccine dose, with a difference favoring the mRNA1273 vaccine. Both vaccines were found to have little effect against any COVID-19 infection over the follow-up period but were found highly effective in preventing substantial symptomatic disease, with possibly important implications at both personal and health policy levels.

## Methods

### Study setting and design

This open-label controlled intervention study with a 180-day follow-up was conducted at the Sheba Medical Centre (SMC), the largest tertiary hospital in Israel. Eligible participants were identified among HCW already enrolled in the Sheba HCW COVID-19 Cohort study^4,9,25,26^, were 18 years of age or older, with no known history of COVID-19 infection, who previously responded to a vaccine dose (i.e., at least one serology assay with IgG > 100 BAU throughout the pandemic), and who received the third dose of the BNT162b2 vaccine at least four months earlier. To enroll persons at higher risk of infection, the trial population was selected among participants who had documented IgG titers below 700 BAU during the 90 days before the beginning of the study (0–90 days prior to vaccination date for each group and their controls, respectively).

The study included four arms: two vaccine arms, BNT162b2 and mRNA1273, and a corresponding control arm for each vaccine arm. Enrollment to the two vaccine arms was time-dependent—those enrolled between December 27–28, 2021, joined the BNT162b2 arm, and those enrolled between January 5–6, 2022, joined the mRNA1273 arm. Age-matched controls (with an age difference of ±5 years) were selected in a 2:1 ratio from the remaining eligible HCW who did not enroll in either vaccine arm. A single control was allowed to serve as a matched control of both intervention groups. Follow-up began at receipt of the vaccine dose in each vaccine arm and its corresponding control arm. This is an interim analysis of 90 ± 14 days of follow-up. Participants were followed until a positive SARS-CoV-2 test or the end of follow-up (April 8, 2022, for the BNT162b2 group and their controls, and April 17, 2022 for the mRNA1273 and their controls) a total of 102 days of follow-up. In the control group, follow-up was additionally terminated if individuals received the fourth dose (on the day of vaccination), which became available to the HCW population on January 2, 2022. The trial took place during the fifth surge of SARS-CoV-2 infections in Israel, which was predominated by the Omicron BA.1 VOC.

The full Study Protocol and Statistical Analysis Plan are included in the Supplementary Information.

### Study conduct

Upon enrollment, following receipt of informed consent, medical and vaccine history, along with blood samples for immunogenicity assays, and nasopharyngeal swabs for SARS-CoV-2 quantitative real-time polymerase chain reaction (qRT-PCR), were collected from individuals in the vaccine arms. The designated vaccine dose was then administered; either 30 μg of BNT162b2, for those enrolled on December 27–28, 2021, or 50 μg of mRNA1273 for those enrolled on January 5–6, 2022.

Planned follow-up encounters took place on 7, 14, 21, 60, and 90 days after receipt of the vaccine dose. Each encounter included screening for symptoms, a SARS-CoV-2 qRT-PCR nasopharyngeal swab, and blood sample collection for immunogenicity assays. In addition, participants were allowed to perform additional serological tests between the planned encounters. During the study period, participants were encouraged to test by either qRT-PCR or an antigen rapid diagnostic test (Ag-RDT) in any case of exposure to an individual with SARS-CoV-2 or the development of symptoms, and at least once weekly. Participants in the control group were similarly encouraged to perform SARS-CoV-2 tests at least once weekly. To enhance compliance, personal telephone reminders were made to all participants. A final assessment of SARS-CoV-2 infection status (either qRT-PCR, Ag-RDT tests, or an uncharacteristic increase in IgG) and symptoms was performed after 102 days by telephone, through electronic questionnaires, and by linkage to the national COVID-19 testing database.

### Variables

The exposure of interest was the type of mRNA SARS-CoV-2 vaccine received as a fourth vaccine dose following a three-dose BNT162b2 regimen: BNT162b2, mRNA1273, or no fourth vaccine dose. The primary outcomes were IgG, neutralizing antibodies, and microneutralization and the secondary outcomes were IgA, T cell activation, and clinical outcomes of SARS-CoV-2 infection and substantial symptomatic disease. The substantial symptomatic disease was defined as two or more days in which the participant was mostly in bed due to feeling unwell.

A complete description of the laboratory methods used for each outcome, including the virus strains information and handling and neutralization assays used, as well as the intervals in which they were measured, is included in the Supplementary Methods. Direct neutralization assays were performed on a randomly selected sample of 25 individuals. SARS-CoV-2 infection was defined as either a positive SARS-CoV-2 qRT-PCR test, a positive Ag-RDT test, or an uncharacteristic increase in IgG after a decline from peak vaccination levels was already observed. All SARS-CoV-2 tests conducted in SMC or in other medical institutions (including community settings) are reported to a central country-wide reporting system, and participants were actively inquired about the results of home rapid antigen tests (via electronic questionnaires or telephone calls). An uncharacteristic increase in IgG was defined as a positive case when an IgG increase of >500 BAU or >1000 BAU was observed from previous IgG titers of <700 or >700, respectively. Similarly, for controls, cases were defined as positive when IgG increased >200 BAU, due to the initially lower titers. All cases were assessed by electronic questionnaires or telephone calls to ascertain disease severity.

A detailed definition of the variables is included in Table S6. We used a self-report questionnaire to determine participants’ sex with a binary option- female or male.

### Statistical analysis

The sample size was chosen to allow the identification of a twofold difference in the GMT of IgG between the two intervention groups, with an alpha of 0.05 and a power of 0.8. Under these conditions, the required sample size for the study was deemed to be 65 participants in each intervention group.

The study population was described using the appropriate summary statistics used for different variables.

### Immunogenicity

For each outcome, levels were plotted as a function of vaccine type (BNT162b2 or mRNA1273) and time following vaccination. An estimate of the geometric mean with 95% confidence intervals at each encounter was overlaid on the plot.

The association between the logarithm of the different outcomes (base 10 logarithm for SARS-CoV-2 IgG and IgA and base 2 logarithm for neutralizing antibodies, and T cell activation), vaccine type, and time, was modeled using a multivariable linear regression adjusted for age, sex, BMI, immunosuppression, and the number of comorbidities. A random intercept was included for each participant. For the purpose of the models, outcome levels were considered constant for the first 30 days following vaccination (peak level) and linearly changing (as a function of time) thereafter. We first modeled each vaccine separately to estimate peak levels (the intercept) and the decay (the coefficient of time elapsed). We then modeled both vaccines together to contrast the decay rates between them (the coefficient of the interaction between vaccine type and time elapsed). In each model, we report the exponentiated coefficient as the Geometric Mean Ratio (GMR). HCW with missing covariates data were rare and were thus dropped from the analysis. Outcome missing data were handled by the inclusion of the random effect, under a missing at random assumption.

### Vaccine efficacy

Individuals who were diagnosed with SARS-CoV-2 during the first seven days of follow-up were excluded from the vaccine efficacy analysis, as the fourth dose was assumed to not yet have an effect at that point. Furthermore, participants from the control groups who received the fourth vaccine dose outside the study settings were censored on the day of vaccination. The Kaplan–Meier estimator was used to plot cumulative incidence curves and to estimate the crude risk for infection and substantial symptomatic disease at 102 days. Multivariable Poisson regression, adjusted for age and sex, was used to estimate the incidence rate ratio (IRR) for infection and substantial symptomatic disease between vaccinated and unvaccinated individuals. VE of receiving a fourth dose compared to receiving three vaccine doses was defined as 1-IRR. This analysis was performed separately for the BNT162b2 and mRNA1273 vaccines. Observations with missing outcome data for substantial symptomatic disease, which were rare, were dropped.

Excel 2016 was used for data collection. Analyses were performed with the use of R software, version 4.0.4. Some figures were drawn using Graphpad Prism Software version 9.0.

### Ethics

The ongoing trial is being conducted in accordance with the International Council for Harmonization of Technical Requirements for Pharmaceuticals for Human Use, Good Clinical Practice guidelines, and applicable government regulations. The national and the institutional review board (Sheba Medical Center, IRB committee) approved the protocol and the consent forms (IRB-8980-21 and IRB-9035-21). All participants provided written informed consent before enrollment. This is an independent study, not sponsored or funded by any commercial company. All trial vaccines were acquired through the government procurement process. Participants were not compensated for participation.

Trial registration numbers (clinicaltrials.gov): NCT05231005 and NCT05230953. The study was approved by the national and institutional review boards in December 2021, and thus we immediately initiated the study and submitted the protocols for clinicaltrials.gov, but due to requested clarifications, the final posting was delayed to February 2022.

### Reporting summary

Further information on research design is available in the Nature Portfolio Reporting Summary linked to this article.

## Supplementary information

Reporting Summary

Supplementary Information

## Data Availability

The de-identified datasets generated during and/ or analyzed during the current study are available from the corresponding author on reasonable request for research purposes only. Requests will be answered within 2–3 business days to understand the research use, and data will be provided within 3–4 weeks. Source data are provided with this paper as source data files. Source data are provided with this paper.

## Source data

Source Data

## Code availability

The analytic code is available on Zenodo (https://zenodo.org/record/7190913#.Y0b1wuzMK3J).
