# Supplementary material for: Immunogenicity and efficacy of fourth BNT162b2 and mRNA1273 COVID-19 vaccine doses; three months follow-up
Source: Nat Commun. 2022 Dec 13;13:7711. doi: 10.1038/s41467-022-35480-2 (PMC9745767; doi:10.1038/s41467-022-35480-2)
Supplement: Supplementary file 1 — Reporting Summary [file 41467_2022_35480_MOESM1_ESM.pdf]

nature research

Corresponding author(s): Gili Regev-Yochay

Last updated by author(s): 11/19/2022

Reporting Summary

Nature Research wishes to improve the reproducibility of the work that we publish. This form provides structure for consistency and transparency in reporting. For further information on Nature Research policies, see our Editorial Policies and the Editorial Policy Checklist.

Statistics

For all statistical analyses, confirm that the following items are present in the figure legend, table legend, main text, or Methods section.

n/a	Confirmed	
☐	☒	The exact sample size (*n*) for each experimental group/condition, given as a discrete number and unit of measurement
☐	☒	A statement on whether measurements were taken from distinct samples or whether the same sample was measured repeatedly
☒	☐	The statistical test(s) used AND whether they are one- or two-sided<br>*Only common tests should be described solely by name; describe more complex techniques in the Methods section.*
☐	☒	A description of all covariates tested
☐	☒	A description of any assumptions or corrections, such as tests of normality and adjustment for multiple comparisons
☐	☒	A full description of the statistical parameters including central tendency (e.g. means) or other basic estimates (e.g. regression coefficient) AND variation (e.g. standard deviation) or associated estimates of uncertainty (e.g. confidence intervals)
☒	☐	For null hypothesis testing, the test statistic (e.g. *F*, *t*, *r*) with confidence intervals, effect sizes, degrees of freedom and *P* value noted<br>*Give P values as exact values whenever suitable.*
☒	☐	For Bayesian analysis, information on the choice of priors and Markov chain Monte Carlo settings
☐	☒	For hierarchical and complex designs, identification of the appropriate level for tests and full reporting of outcomes
☒	☐	Estimates of effect sizes (e.g. Cohen's *d*, Pearson's *r*), indicating how they were calculated

Our web collection on statistics for biologists contains articles on many of the points above.

Software and code

Policy information about availability of computer code

Data collection	Excel 2016
Data analysis	Analyses were performed with the use of R software, version 4.0.4. Some figures were drawn using Graphpad Prism Software version 9.0. The analytic code is available on Zenodo (https://zenodo.org/record/7190913#.Y0b1wuzMK3J).

For manuscripts utilizing custom algorithms or software that are central to the research but not yet described in published literature, software must be made available to editors and reviewers. We strongly encourage code deposition in a community repository (e.g. GitHub). See the Nature Research guidelines for submitting code & software for further information.

Data

Policy information about availability of data

All manuscripts must include a data availability statement. This statement should provide the following information, where applicable:

- Accession codes, unique identifiers, or web links for publicly available datasets
- A list of figures that have associated raw data
- A description of any restrictions on data availability

The de-identified datasets generated during and/ or analyzed during the current study are available from the corresponding author on reasonable request for research purposes only. Requests will be answered within 2-3 business days to understand the research use, and data will be provided within 3-4 weeks. Source data for figures 2, 3, and 4 are provided with this paper as source data files.

Field-specific reporting

Please select the one below that is the best fit for your research. If you are not sure, read the appropriate sections before making your selection.

[x] Life sciences [ ] Behavioural & social sciences [ ] Ecological, evolutionary & environmental sciences

For a reference copy of the document with all sections, see nature.com/documents/nr-reporting-summary-flat.pdf

Life sciences study design

All studies must disclose on these points even when the disclosure is negative.

Sample size	The sample size was chosen to allow identification of a 2-fold difference in the GMT of IgG between the two intervention groups, with an alpha of 0.05 and power of 0.8. Under these conditions, the required sample size for the study was deemed to be 65 participants in each intervention group.
Data exclusions	No data was excluded.
Replication	Not Applicable. Only one sample per time point per individual can be obtained on this prospective study
Randomization	Randomization was not used. Enrollment to the two vaccine arms was time-dependent – those enrolled by December 28, 2021, joined the BNT162b2 arm, and those enrolled later, until January 6, 2022, joined the mRNA1273 arm. Age-matched controls (with an age difference of ±5 years) were selected in a 2:1 ratio from the remaining eligible HCW who did not enroll in either vaccine arm.
Blinding	Not applicable. This is an interventional study but not an RCT. Additionally, the study originated as two different studies with two different IRBs approvals for each vaccine given to our HCW

Reporting for specific materials, systems and methods

We require information from authors about some types of materials, experimental systems and methods used in many studies. Here, indicate whether each material, system or method listed is relevant to your study. If you are not sure if a list item applies to your research, read the appropriate section before selecting a response.

Materials & experimental systems

n/a	Involved in the study	
[ ]	[x]	Antibodies
[ ]	[x]	Eukaryotic cell lines
[x]	[ ]	Palaeontology and archaeology
[x]	[ ]	Animals and other organisms
[ ]	[x]	Human research participants
[ ]	[x]	Clinical data
[x]	[ ]	Dual use research of concern

Methods

n/a	Involved in the study	
[x]	[ ]	ChIP-seq
[x]	[ ]	Flow cytometry
[x]	[ ]	MRI-based neuroimaging

Antibodies

Antibodies used	Goat anti-human IgG horseradish peroxidase (HRP) conjugate (Jackson ImmunoResearch, PA, USA Code:109-035-088) (diluted 1:15000). Mouse anti-human IgA HRP conjugate (Abcam, MA, USA, product number: ab7383) (diluted 1:2000).
Validation	Validation for both IgG and IgA was preformed by manufacture and also during the development of the assay (PMID: 33227020).

Eukaryotic cell lines

Policy information about cell lines

Cell line source(s)	Vero cells derived from African green monkey were obtained from ATCC. Expi293 cells were obtained from ThermoFisher (Cat#A14527).
Authentication	By ATCC and regularly by assessment of the characteristics morphology and growth rate in culture. By ATCC and ThermoFisher and regularly.
Mycoplasma contamination	Cells were tested negative for mycoplasma contamination
Commonly misidentified lines (See ICLAC register)	None

Human research participants

Policy information about studies involving human research participants

Population characteristics	Eligible participants were identified among HCW already enrolled in the Sheba HCW COVID-19 Cohort study (monthly serological follow-up), were 18 years of age or older, with no known history of COVID-19 infection, who previously responded to a vaccine dose (i.e., at least one serology assay with IgG >100 BAU), and who received the third dose of the BNT162b2 vaccine at least four months earlier. To enroll persons at higher risk of infection, the trial population was selected among participants who had documented IgG titers below 700 BAU from the 90 days before the beginning of the study. We used self-report to determine participants sex with binary option- female or male.
Recruitment	HCW from the Sheba HCW COVID-19 Cohort study who met the inclusion criteria were invited to participate in the study. A computer-based questionnaire about demographic characteristics and coexisting conditions was sent electronically to all participants. All analysis were deidentified.
Ethics oversight	The national and institutional (Sheba Medical Center, IRB committee) review boards approved the protocol and the consent forms. Participants were not compensated for participation.

Note that full information on the approval of the study protocol must also be provided in the manuscript.

Clinical data

Policy information about clinical studies

All manuscripts should comply with the ICMJEguidelines for publication of clinical research and a completedCONSORT checklist must be included with all submissions.

Clinical trial registration	NCT05231005 and NCT05230953
Study protocol	The study protocol is attached to the manuscript.
Data collection	This is an open-labled, controlled intervention trial. Two intervention arms are planned and participants will be designated to each arm on a time dependent manner. Eligible participants are those enrolled to the Sheba COVID-19 Cohort study (IRB 8008-20), who have been followed for serology testing, had an available test from 60 days prior recruitment with a result of IgG of 700BAU or below (i.e., under 40 percentile of the full cohort on Dec 2021) and have received the third BNT162b2 dose at least 4 months earlier. Initially volunteers were recruited and designated to the BNT162b2 arm (during December 27-28, 2021), and once approval of the second arm was received, volunteers were recruited and designated to the second – mRNA1273 arm (during January 5-6, 2022). Age matched controls, who are eligible to be enrolled to the intervention arm will be selected for the efficacy analysis. Planned follow-up encounters took place on 7, 14, 21, 60, and 90 days post-vaccination. All data was collected within the Sheba Medical Center, between December 27, 2021, to April 17, 2022.
Outcomes	The primary outcomes: SARS-CoV-2 RBD-IgG, Pseudoneutralization, microneutralizaion of Delta, Omicron and WT strains, were measured to assess immune response to the 4th COVID-19 mRNA vaccines. IgG and Pseudoneutralization were measured in BAU at each time point, and microneutralizaion 14 and 90 days post-vaccination.<br>The secondary outcomes: IgA and T cells activity were measured to assess immune response to the 4th COVID-19 mRNA vaccines, and infected cases were assessed for PCR or Rapid Ag test positivity and COVID-19 symptoms to evaluate breakthrough infection incidence and characterize cases. IgA titers were measured in sample-to-cutoff ratio (s/co) 0, 14, and 90 days post-vaccination, T cell activity was measured 0, 14, 60, and 90 days post-vaccination. COVID-19 tests were taken upon each visit and weekly when the pandemic surge was high (>10k newly detected cases/d). COVID-19 related symptoms were evaluated in each visit and for all positive cases.
