# Supplementary material for: Immunogenicity and efficacy of fourth BNT162b2 and mRNA1273 COVID-19 vaccine doses; three months follow-up
Source: Nat Commun. 2022 Dec 13;13:7711. doi: 10.1038/s41467-022-35480-2 (PMC9745767; doi:10.1038/s41467-022-35480-2)
Supplement: Supplementary file 2 — Supplementary Information [file 41467_2022_35480_MOESM2_ESM.pdf]

Supplementary material

This appendix has been provided by the authors to give readers additional information about their work.

Supplementary Materials

Table of Contents

Supplementary Methods S1- Immunogenicity 2

Supplementary Methods S2- SARS-CoV-2 testing 4

Supplementary Table S1- Baseline characteristics of participants followed by intervention group 5

Supplementary Table S2- Crude values of serological markers 6

Supplementary Table S3- Immune response adjusted models 7

Supplementary Table S4- Infection and substantial disease cumulative incidence 9

Supplementary Table S5- Vaccine efficacy Poisson regression model 10

Supplementary Table S6- Variable definitions 11

Authors list: Michal Canetti[1,2], Noam Barda[3,4,5], Mayan Gilboa[1,2], Victoria Indenbaum[2,6], Michal Mandelboim[2,6], Tal Gonen[1,2], Keren Asraf[2,7], Yael Weiss-Ottolenghi[1,2], Sharon Amit[2,8], Ram Doolman[2,7], Ella Mendelson[2,6], Dror Harats[9], Laurence S Freedman[10], Yitshak Kreiss[2,9], Yaniv Lustig[2,6] and Gili Regev-Yochay[1,2]

Supplementary Methods S1- Immunogenicity

SARS-CoV-2 IgG II Quant (Abbott, IL, USA)

Samples were centrifuged at room temperature, at 4000g, for 4 minutes. Serum was tested for IgG antibodies against the SARS-COV-2 spike RBD ancestral strain using the commercial automatic chemiluminescent microparticle immunoassay (CMIA) SARS-CoV-2 IgG II Quant (Abbott, IL, USA) according to the manufacturer's instructions. Following a conversion factor between AU/mL and the WHO binding antibody units (BAU/mL) which have been established as 1 BAU = 0.142*AU by the manufacturer, all IgG levels are given in BAU.

SARS-CoV-2 Pseudovirus (psSARS-2) Neutralization Assay

SARS-CoV-2 Pseudovirus (psSARS-2) Neutralization Assay was performed using a propagation-competent vesicular stomatitis virus encoding the ancestral strain of SARS-CoV-2 spike protein as previously published (1). The Pseudovirus Neutralization Assay was validated against the WHO international units (IU) and was also shown to be highly correlative to authentic SARS-CoV-2 virus micro-neutralization assay. Following titration, 100 focus forming units (FFU) of psSARS-2 were incubated with 2-fold serial dilution of heat inactivated (56°C for 30 min) tested sera. After incubation for 60 min at 37°C, virus/serum mixture was transferred to Vero E6 cells that have been grown to confluence in 96-well plates and incubated for 90 min at 37°C. After the addition of 1% methyl cellulose in dulbecco's modified eagle's medium (DMEM) with 2% of fetal bovine serum (FBS), plates were incubated for 24 hours, and 50% plaque reduction titer was calculated by counting green fluorescent foci using a fluorescence microscope (EVOS M5000, Invitrogen). Sera not capable of reducing viral replication by 50% at 1 to 16 dilution or below were considered non-neutralizing. For clear presentation, non-neutralizing samples were marked as a titer of 2.

Live Micro-neutralization assay

VERO-E6 cells at concentration of 20*103/well were seeded in sterile 96-wells plates with 10% FCS MEM-EAGLE medium and stored at 37ºC for 24 hours. One hundred TCID50 of wild type, Delta, and Omicron BA.1 and Ba.2 SARS-CoV-2 isolates (isolated from SARS-CoV-2 positive individuals as described previously (2)) were incubated with inactivated sera diluted 1:8 to 1:16,384 in 96 well

plates for 60 minutes at 33°C. Virus-serum mixtures were added to the Vero E-6 cells and incubated for five days at 33°C after which Gentian violet staining (1%) was used to stain and fix the cell culture layer. Neutralizing dilution of each serum sample was determined by identifying the well with the highest serum dilution without observable cytopathic effect. A dilution equal to 1:10 or above was considered neutralizing.

IgA assay

IgA antibodies against the S1 domain of the spike protein of SARS-CoV-2 (expressed recombinantly in the human cell line HEK 293) were detected by a semiquantitative enzyme-linked immunoassay (ELISA) (anti-SARS-CoV-2 ELISA IgA, Euroimmun, Lübeck, Germany). Samples were tested according to the manufacturer's instructions. For the ELISA, a 96 well microtiter Polysorb plate (Nunc, Thermo, Denmark) was coated overnight at 4°C with 50µl per well of 2µg/ml. After blocking with 5% skimmed milk at 25°C for 60 minutes, positive and negative control and human serum samples without replications and calibrator (sample at the cut-off O.D value) in triplicate (all diluted 1:100 with 3% skimmed milk), were added to antigen coated wells. The plate was incubated at 25°C for 120 minutes, washed and a HRP-conjugated isotype specific antibody (anti-human IgA horseradish peroxidase (HRP) conjugate (Abcam, MA, USA, product number: ab7383) (diluted 1:2000)) was added to each well for 60 min. After washing, incubation of TMB Substrate Solution (Abcam) for 5 min and the addition of stop solution (2N HCl), the OD of each well was measured at 450nm using a micro-plate reader (Sunrise, Tecan).

ELISA index value was defined as the ratio between sample and cut-off optical densities (OD). ELISA index value below 0.9 was considered negative, between 0.9 and 1.1, borderline and equal or above 1.1, positive.

T cell activation

Interferon-γ (IFN-γ)-secreting T cells were enumerated using Elispot IFN-γ kits (IFN-γ kit, ELSP5000/5500, AID Autoimmun Diagnostika) according to manufacturer instructions. For antigen stimulation, 50 µl SARS-CoV-2 peptide pools (S-complete, 130–127–953, Miltenyi Biotech) were used. Test medium was used as a negative control, and phytohaemagglutinin (Mabtech) was used as

a positive control. IFN-γ-secreting cell frequency was quantified using a fluorospot reader (AID Autoimmun Diagnostika). The unspecific background (mean spot-forming units from negative control wells) was subtracted from experimental readings.

Supplementary Methods S2- SARS-CoV-2 Testing

Nasopharyngeal swabs were placed in 3mL of universal transport medium (UTM) or viral transport medium (VTM).

PCR tests were performed according to the manufacturer's instructions using Allplex™ 2019-nCoV (Seegene, S. Korea) platform. Rapid antigen tests were performed according to manufacturer's instructions using STANDARD Q COVID-19 Ag (SD BIOSENSOR, S. Korea).

Cases were defined as positive also when an uncharacteristic increase in IgG levels was observed. We previously reported a substantial increase in IgG levels following vaccination or infection which was maintained for 30 days, after which a slow consistent decline was observed. Therefore, an uncharacteristic increase in IgG levels of >500 BAU or >1000 BAU in HCW with previous IgG results of <700 or >700, respectively was considered as a sero-response due to SARS-CoV-2 infection (3-5).

Supplementary Table S1- Baseline characteristics of participants followed by intervention group

		BNT162b2	Control (BNT162b2)	mRNA1273	Control (mRNA1273)
Participants followed	N	150	297	115	129
Female sex	N (%)	89 (59)	209 (70)	79 (69)	107 (83)
Male sex	N (%)	61 (41)	88 (30)	36 (31)	22 (17)
Age	Median (range)	60.85 (30.44-85.31)	60.77 (30.27-89.61)	55.89 (29.26-86.94)	51.31 (29.21-79.58)
BMI (kg/m²)	Median (IQR)	25.97 (23.03-28.4)	25.4 (23.09-28.08)	24.55 (22.3-28.32)	25.22 (22.15-28.08)
Missing data	%	9		11	
Comorbidities					
0	N (%)	91 (61)	186 (63)	77 (67)	80 (62)
1	N (%)	38 (25)	50 (17)	28 (24)	26 (20)
≥2	N (%)	21 (14)	61 (20)	10 (9)	23 (18)
Missing data	%	9		8	
Hypertension	N (%)	40 (27)	30 (12)	21 (18)	7 (7)
Diabetes	N (%)	13 (9)	17 (7)	12 (11)	3 (3)
Lung disease	N (%)	7 (5)	8 (3)	7 (6)	3 (3)
Heart disease	N (%)	11 (7)	7 (3)	6 (5)	3 (3)
Liver disease	N (%)	1 (0.7)	1 (0.5)	1 (1)	1 (1)
Chronic kidney disease	N (%)	1 (0.7)	1 (0.5)	3 (3)	1 (1)
Autoimmune disease	N (%)	12 (8)	20 (8)	4 (4)	11 (10)
Immunosuppression	N (%)	3 (2)	3 (1)	2 (2)	1 (1)
Missing data	%	9		8	
Profession					
Physician	N (%)	40 (27)	47 (16)	33 (29)	10 (8)
Nurse	N (%)	33 (22)	75 (25)	21 (18)	54 (42)
Allied health professions	N (%)	33 (22)	58 (20)	34 (30)	19 (14)
Administration or maintenance	N (%)	42 (28)	117 (39)	24 (21)	46 (36)
Other personnel	N (%)	2 (1)	0	3 (2)	0

BMI – body mass index.

Supplementary Table S2- Crude values of serological markers

A. IgG titers				
	mRNA1273		BNT162b2	
	N	GMT (95% CI)	N	GMT (95% CI)
Day 0	117	336<br>(286-396)	149	324<br>(291-362)
Day 7	107	2344<br>(1908-2878)	142	1446<br>(1234-1694)
Day 14	98	3527<br>(2957-4207)	136	2952<br>(2573-3387)
Day 21	74	3531<br>(2916-4276)	129	2677<br>(2346-3054)
Day 60	70	1827<br>(1488-2244)	92	1488<br>(1302-1702)
Day 90	56	1442<br>(1194-1741)	68	854<br>(738-989)
B. Neutralizing antibody titers				
	mRNA1273		BNT162b2	
	N	GMT (95% CI)	N	GMT (95% CI)
Day 0	116	337<br>(276-411)	149	425<br>(355-508)
Day 7	107	3357<br>(2648-4256)	142	2709<br>(2200-3335)
Day 14	97	5580<br>(4381-7107)	136	3737<br>(3119-4478)
Day 21	74	3064<br>(2273-4129)	128	3577<br>(3014-4247)
Day 60	69	1515<br>(1136-2020)	93	1032<br>(863-1233)
Day 90	33	1046<br>(772-1417)	25	347<br>(238-507)
C. IgA titers				
	mRNA1273		BNT162b2	
	N	GMT (95% CI)	N	GMT (95% CI)
Day 0	114	1.1<br>(0.98-1.23)	149	0.95<br>(0.85-1.06)
Day 14	97	4.63<br>(3.88- 5.53)	134	3<br>(2.57-3.53)
Day 90	56	1.82<br>(1.48- 2.25)	68	1.39<br>(1.16-1.65)
D. T cells				
	mRNA1273		BNT162b2	
	N	GMT (95% CI)	N	GMT (95% CI)
Day 0	54	6<br>(2-14)	56	4<br>(2-12)
Day 14	35	52<br>(20-134)	52	8<br>(3-22)
Day 60	26	18<br>(5-61)	24	14<br>(3-65)
Day 90	27	11<br>(3-48)	11	11<br>(1-142)

Immunoglobulin G (IgG) and neutralizing antibody titers are presented in binding antibody units (BAU), Immunoglobulin A (IgA) is presented as sample-to-cutoff (s/co) ratio, T cells are presented as activated T cells per 10^6 peripheral blood mononuclear cells (PBMC).

Supplementary Table S3- Immune response adjusted models

Table S3a. Mixed model analysis of variables associated with IgG, neutralizing antibody and IgA titers, and number of activated T cells after receipt of the fourth vaccine dose. The two vaccine types were modeled separately.

	**IgG (95% CI)**	**Neutralizing antibodies (95% CI)**	**IgA (95% CI)**	**T cells (95% CI)**
**A. mRNA1273 model**				
Age	0.99 (0.98-1.01)	0.99 (0.97-1)	1 (0.99-1.01)	0.97 (0.92-1.02)
Sex				
Female	-ref-	-ref-	-ref-	-ref-
Male	0.9 (0.64-1.26)	0.67 (0.43-1.03)	0.98 (0.69-1.4)	0.58 (0.13-2.58)
BMI (kg/m^2)	1.02 (0.98-1.05)	1.03 (0.99-1.08)	1 (0.96-1.03)	0.99 (0.87-1.13)
Number of comorbidities	0.93 (0.75-1.16)	0.95 (0.71-1.27)	1.47 (1.16-1.87)	1.92 (0.67-5.45)
Immunosuppression	0.04 (0.01-0.19)	0.01 (0-0.04)	0.03 (0.01-0.15)	NA*
Decay per week	0.89 (0.88-0.9)	0.79 (0.76-0.82)	0.9 (0.88-0.92)	0.9 (0.77-1.05)
**B. BNT162b2 model**				
Age	1 (1-1.01)	1 (0.99-1.01)	1 (0.99-1.02)	0.97 (0.91-1.05)
Sex				
Female	-ref-	-ref-	-ref-	-ref-
Male	0.91 (0.71-1.17)	0.96 (0.72-1.28)	0.96 (0.71-1.3)	1.35 (0.21-8.79)
BMI (kg/m^2)	1.01 (0.99-1.04)	1 (0.98-1.03)	1.01 (0.98-1.04)	0.96 (0.82-1.13)
Number of comorbidities	0.96 (0.82-1.11)	0.96 (0.8-1.14)	1.06 (0.89-1.28)	0.97 (0.35-2.69)
Immunosuppression	0.08 (0.03-0.19)	0.27 (0.09-0.79)	0.4 (0.13-1.2)	0.01 (0-29.17)
Decay per week	0.86 (0.86-0.87)	0.74 (0.72-0.76)	0.92 (0.9-0.93)	0.9 (0.74-1.09)

Number of comorbidities was considered a continuous variable. BMI- body mass index. *There was insufficient data to estimate the coefficient for the Immunosuppression parameter in this model.

Table S3b. Mixed model analysis of variables associated with IgG, neutralizing antibody and IgA titers, and number of activated T cells after receipt of the fourth vaccine dose. The two vaccines were modeled together.

	IgG (95% CI)	Neutralizing antibodies (95% CI)	IgA (95% CI)	T cells (95% CI)
Age	1 (0.99-1.01)	0.99 (0.98-1)	1 (0.99-1.01)	0.97 (0.93-1.01)
Sex				
Female	-ref-	-ref-	-ref-	-ref-
Male	0.9 (0.74-1.1)	0.81 (0.63-1.05)	0.98 (0.78-1.23)	0.87 (0.27-2.8)
BMI (kg/m^2)	1.01 (1-1.03)	1.01 (0.99-1.04)	1 (0.98-1.03)	0.97 (0.88-1.08)
Number of comorbidities	0.94 (0.83-1.06)	0.89 (0.76-1.04)	1.16 (1.01-1.34)	1.21 (0.6-2.46)
Immunosuppression	0.06 (0.03-0.13)	0.06 (0.02-0.16)	0.19 (0.08-0.48)	0.01 (0-10.56)
Peak levels				
mRNA1273 peak	-ref-	-ref-	-ref-	-ref-
BNT162b2 peak	0.9 (0.75-1.1)	0.94 (0.72-1.22)	0.71 (0.57-0.89)	0.46 (0.14-1.55)
mRNA1273 multiplicative decay per week	0.89 (0.88-0.9)	0.79 (0.77-0.82)	0.9 (0.88-0.91)	0.89 (0.76-1.04)
Additional multiplicative decay per week in the BNT162b2 group*	0.98 (0.96-0.99)	0.93 (0.89-0.98)	1.02 (1-1.05)	1.03 (0.8-1.32s)

Number of comorbidities was considered a continuous variable. BMI- body mass index.
* This parameter was modeled using an interaction term between vaccine type and time (in weeks).

Supplementary Table S4- Infection and substantial disease cumulative incidence

	Incidence<br>(95%, CI)
A. Cumulative incidence of infection	
mRNA1273 controls<br>(N=129)	0.5 (0.4-0.58)
mRNA1273<br>(N=115)	0.39 (0.3-0.47)
BNT162b2 controls<br>(N=297)	0.48 (0.39-0.55)
BNT162b2<br>(N=150)	0.44 (0.36-0.51)
B. Cumulative incidence of substantial disease	
mRNA1273 controls<br>(N=126)	0.2 (0.11-0.27)
mRNA1273<br>(N=115)	0.03 (0-0.06)
BNT162b2 controls<br>(N=291)	0.23 (0.15-0.3)
BNT162b2<br>(N=147)	0.09 (0.03-0.13)

Supplementary Table S5- Vaccine efficacy Poisson regression model

	mRNA1273<br>IRR (95%, CI)	BNT162b2<br>IRR (95%, CI)
A. Infection		
Vaccination status		
Controls	-ref-	-ref-
Fourth dose vaccine	0.75<br>(0.51-1.11)	0.97<br>(0.69-1.38)
Age	0.97<br>(0.96-0.99)	0.97<br>(0.96-0.98)
Sex male	0.93<br>(0.58-1.44)	1.24<br>(0.85-1.78)
B. Substantial disease		
Vaccination status		
Controls	-ref-	-ref-
Fourth dose vaccine	0.11<br>(0.02-0.37)	0.29<br>(0.13-0.57)
Age	0.96<br>(0.92-1)	0.97<br>(0.95-0.99)
Sex male	0.38<br>(0.06-1.26)	1.16<br>(0.57-2.21)

Supplementary Table S6- Variable definitions

Variable	Values	Definition
A. Exposure		
Vaccine type	BNT162b2/ mRNA1273	Vaccine type received upon enrollment
B. Immunological outcomes		
IgG	Numeric	Anti-RBD IgG GMT tested on days 0, 7, 14, 21, 60, and 90
Neutralizing antibodies	Numeric	Neutralizing antibodies GMT on days 0, 7, 14, 21, 60, and 90
IgA	Numeric	Anti-RBD IgA GMT on days 0, 14, and 90
Direct neutralization titers	Numeric	Direct neutralization levels against the Index virus, Delta, Omicron BA.1, and Omicron BA.2 variants of concern on days 14 and 90
Number of activated T cells	Numeric	Number of activated T cells GMT on days 0, 14, 60, and 90
C. Clinical outcomes		
SARS-CoV-2 infection	0/1	Positive SARS-CoV-2 qRT-PCR test, a positive Ag-RDT, or an uncharacteristic increase in IgG levels*
SARS-CoV-2 substantial disease	0/1	Positive SARS-CoV-2 participant who spent two or more days mostly in bed due to feeling unwell
D. Covariates		
Age	Numeric	Participant's age upon enrollment
Sex	Female/ male	Participant's sex based on self-report
BMI	Numeric	BMI calculated as weight/m^2 upon enrollment
Immunosuppression	0/1	Including organ transplant recipient, currently undergoing biological therapy or chemotherapy, treated with corticosteroids, underwent splenectomy, or diagnosed with HIV
Number of comorbidities	0, 1, +2	Including hypertension, dyslipidemia, autoimmune disease, diabetes, heart disease, lung disease, coagulation disorder, liver disease, and kidney disease

IgG- anti-RBD immunoglobulin G, IgA- anti-RBD immunoglobulin A, GMT- geometric mean titers, SARS-CoV-2- severe acute respiratory syndrome coronavirus 2, qRT-PCR- quantitative real-time polymerase chain reaction, Ag-RDT- antigen rapid diagnostic test, BMI- body mass index, HIV- human immunodeficiency virus. *Uncharacteristic increase in IgG levels was defined as >500 BAU or >1000 BAU in HCW with previous IgG results of <700 or >700, respectively.

Supplementary references

1) Lustig, Y. *et al.* BNT162b2 COVID-19 vaccine and correlates of humoral immune responses and dynamics: a prospective, single-centre, longitudinal cohort study in health-care workers. *Lancet Respir. Med.* **9**, 999–1009 (2021).
2) Nemet, I. *et al.* Third BNT162b2 Vaccination Neutralization of SARS-CoV-2 Omicron Infection. *N. Engl. J. Med.* **386**, 492–494 (2022).
3) Gilboa, M. *et al.* Early Immunogenicity and Safety of the Third Dose of BNT162b2 Messenger RNA Coronavirus Disease 2019 Vaccine Among Adults Older Than 60 Years: Real-World Experience. *J. Infect. Dis.* **225**, 785–792 (2022).
4) Lustig, Y. *et al.* Superior immunogenicity and effectiveness of the third compared to the second BNT162b2 vaccine dose. *Nat. Immunol.* **23**, 940–946 (2022).
5) Regev-Yochay, G. *et al.* Efficacy of a Fourth Dose of Covid-19 mRNA Vaccine against Omicron. *N. Engl. J. Med.* **386**, 1377–1380 (2022).

Study Protocol

Immunogenicity of a Fourth mRNA COVID-19 vaccine dose;

Homologous (4 doses of BNT162b2) (IRB-8980-21) or heterologous (mRNA1273 following 3 doses of BNT162b2) (IRB-9035-21)

Background: The Omicron variant of concern (VOC) is currently rapidly spreading worldwide, with extremely high transmission rates, with an estimated R of >3 (Barnard et al., n.d.; Khoury et al., n.d.) . While the virulence of this VOC is yet unknown, even with a much lower virulence than the delta strain, with such high transmission, once again the pandemic will have severe effects on healthcare capacity. Thus, further effort to contain this VOC is required. Currently, the only successful key in containment of the SARS-CoV-2 pandemic was vaccination. Following the first two doses, extremely high vaccine effectiveness was reported from many real-world observational studies(Amit et al., 2021; Dagan et al., 2021; Haas et al., 2021). Several vaccines were reported with high vaccine efficacy and effectiveness, mostly, the mRNA vaccines, of which the BNT162b2 was rolled out in Israel. Yet, with time, waning of this effect was reported, both in immunogenicity as well as in vaccine effectiveness against infections and hospitalizations (Bar-On et al., 2021; Levin et al., 2021)

Following these reports, Israel initiated rollout of the third dose of BNT162b2 on July 29, initially to adults 60 years and older and within a few weeks expanded it to the whole population if at least 5 months have passed from the second dose. We have recently reported the early effects of the 3rd dose(Gilboa et al., 2021) , showing significant increase in IgG and Neutralizing antibodies, as well as increased T-cell activity in a small group of non-responders to first two doses. We have also submitted further data, showing the superiority of the third dose in aspects of immunogenicity, with significantly higher amounts of antibodies as well as improved quality as measured by higher avidity and neutralizing capacity (see yet unpublished data Appendix A1).

We now have preliminary, yet unpublished data, showing slow waning of the third dose immune response within 4 months after the third dose (Appendix A2). Simultaneously, Milo R. et al presented yet unpublished data on early waning of vaccine effectiveness of the third dose. While these data would not have been worrisome in the Delta VOC era, this may be different with the emergence of the Omicron VOC.

It was recently reported, by us and others that vaccine effectiveness against the Omicron VOC is decreased. This was shown by reduced neutralization efficiency of Omicron VOC compared to that of the Delta VOC or the wild type (Wu-1), by sera of vaccinated individuals. We have just reported (Nemet et al., n.d.) , accepted to NEJM) that sera from BNT162b 2-dose vaccinated individuals does not neutralize Omicron at all, while it neutralizes to a low level the Delta VOC and more so the Wu-

1. We compared this to 3-dose vaccinated, and also showed a significantly reduced neutralization of early 3-dose vaccinated as compared to the efficiency against Wu-1 or the delta VOC. Furthermore, data from Germany showed that neutralization of Omicron decreases within 3 months following the 3rd dose.

These data raise the question of when and will we need a 4th dose to cope with the emergence of Omicron. However, if we have reached the maximal effect of the current vaccine against Omicron, with a third dose, will a 4th dose have any added value? In this respect, several studies have demonstrated improved immunogenicity of 1 vaccine dose to SARS-CoV-2 recovered patients, yet a 2nd dose to these patients had very little added value. It is thus unclear whether a 4th dose of the current vaccine, which is not directed specifically at the Omicron VOC, can further improve our immunity and protection from this strain, and will it have any added value.

The Sheba HCW COVID Cohort was established on April 2020, when HCW (including employees, students, volunteers and retired personnel), were recruited to a large serology longitudinal follow up study. Participation in the study is confidential and is not disclosed to the worker's direct supervisor. Since then, nearly 7000 HCW who were vaccinated by BNT162b2 are being followed monthly with blood sampling for immune responses, including mostly IgG, but also Neutralizing ab, avidity, microneutralization, IgA and cellular activation. Through this cohort we found the correlation between neutralizing ab levels and SARS-CoV-2 infections (Bergwerk et al., 2021) , the waning of immunity (Levin et al., 2021)and more. In our study, the median neutralizing ab titer of cases was 190, while it was significantly higher (530) among controls. Furthermore, (*Immune Correlates Analysis of the MRNA-1273 COVID-19 Vaccine Efficacy Clinical Trial*, n.d.) report that titer of 1000 or more are correlated with 96% vaccine efficacy and a titer of 100 is correlated with 91%, but this is for the era before Omicron. We and others have shown a decreased efficacy for Omicron of 8-fold to 24-fold compared to WT.

Here, we will study the potential immunogenicity of a 4th dose, together with assessing safety and efficacy in preventing infections.

METHODS:

Study Design

This is an open-labled, controlled intervention trial. Two intervention arms are planned and participants will be designated to each arm on a time dependent manner. Eligible participants are those enrolled to the Sheba COVID-19 Cohort study (IRB 8008-20), who have been followed for serology testing, have an available test from the recent 60 days with a result of IgG of 700BAU or below (i.e., under 40 percentile of the full cohort on Dec 2021) and have received the third BNT162b2 dose at least 4 months earlier (see below full inclusion/exclusion criteria). Initially volunteers will be designated to the BNT162b2 arm, and once approval of the

second arm will be received, volunteers will be designated to the second – mRNA1273 arm. Age matched controls, who are eligible to be enrolled to the intervention arm will be selected for the efficacy analysis.

Figure 1: Flow Chart of eligibility and study population selection

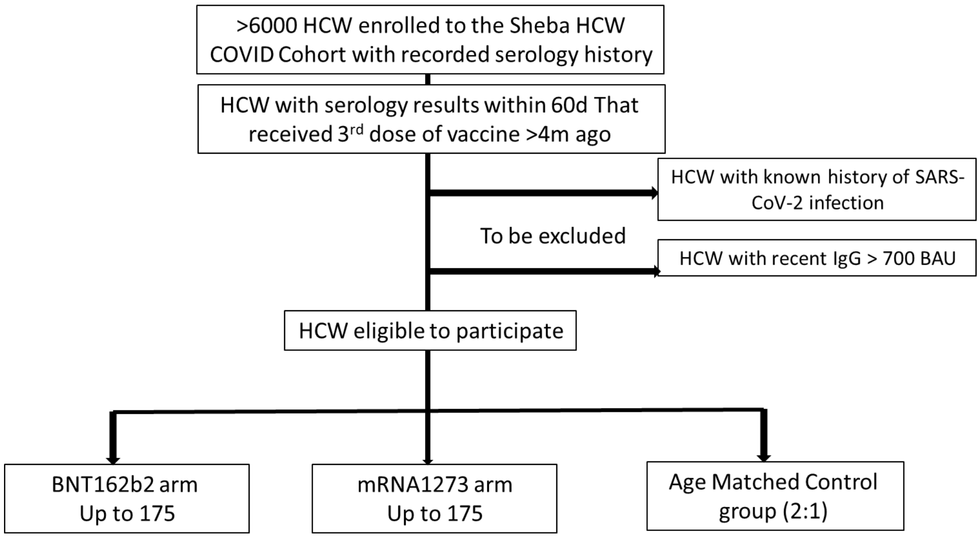

Inclusion Criteria:

1. Age: Volunteer must be at least 18 years of age, at the time of signing the informed conset.
2. Sex: Male or Female.
3. Received 3 doses of BNT162b2 with the 3rd dose at least 4 months previously.
4. Have a serology test within the previous 3 months of 700 BAU or less.
5. Responded to the previous vaccine doses, i.e. at least one IgG>100.
6. Medical Conditions: Volunteers with any medical condition are allowed, as long as they adhere to the criteria above.
7. Agreed to attend all visits and signed the informed consent

Exclusion Criteria:

1. Had previous SARS-CoV-2 infection (detected by either PCR, anti-S IgG before the 1st vaccine dose, anti-N IgG at any stage).
2. Had an allergic response to any of the previous BNT162b2 doses.
3. Had history of myopericarditis.
4. Reported that they do not feel well or have a fever on the day of vaccination.
5. Pregnant on day of recruitment.

Criteria for premature cessation of the study:

- Increased rates of immediate adverse events, higher than those reported in previous studies (11 per million) https://www.cdc.gov/mmwr/volumes/70/wr/mm7002e1.htm)

On recruitment, volunteer:

1. Receive a detailed explanation and signed the informed consent form
2. Filled an initial inclusion/ exclusion criteria questionnaire.
3. Filled a general comorbidity questionnaire, additionally they were screened for COVID-19 symptoms such as fever, cough, anosmia
4. Had 40cc blood drawn for all serology and cellular immunity tests.
5. Performed a PCR for SARS-CoV-2 test
6. Receive the 4th dose of BNT162b2 30μg or mRNA1273 50 μg
7. Had a physician checkup and followup for 15 minutes after receiving the dose.

Four additional visits followed as described in the research timeline:

Research Timeline:

Visit number		1	2	3	4	5	6	7
	Day	0	7	14	21	60	90	180
Vaccine	BNT162b2 30 μg or mRNA1273 50 μg	X						
Blood sample	IgG	X	X	X	X	X	X	X
	PNT	X	X	X	X	X	X	X
	microneutralization	X	X	X	X	X	X	X
	IgA	X	X	X	X	X	X	X
	Tcell activity	X		X		X		X
	Bcell repertoir	X		X		X		X
Nasal/NP swab	SARS-CoV-2 PCR	X	X	X	X	X	X	X
Questionnaires	Background comorbidity	X						
	Adverse events			X		X		

Primary outcomes:

Serology tests as detailed below, including IgG, pseudoneutralization, microneutralization and avidity. These outcomes were compared between pre- and post- 4th dose as well as with those outcomes in the second intervention arm and in the control group.

Adverse event reporting of vaccinated individuals by an electronic survey that will be on day 5 and will be repeatedly sent from visit 2. Serious adverse events will be followed by active surveillance, on each visit, and by direct contact of the research team if a visit has been missed. Serious AE are defined as any adverse event that

resulting in death, hospitalization, permanent damage, requirs treatment in the emergency room or life threatening.

Secondary outcomes:

T-cell activity as in detailed methods appendix.

SARS-CoV-2 cumulative incidence and vaccine efficacy will be assessed, by active surveillance of weekly SARS-CoV-2 PCR testing regardless of any symptom. Additionally, symptoms will be assessed on all visits and participants will be encouraged to perform home antigen testing if any symptom occurs in between PCR testing, or upon exposure to SARS-CoV-2 detected individuals.

All data regarding participants would be stored in secured computers and would be available only for this specific study purpose. Any data transferred to a third party will be de-identified, the identification key will be kept by the PI.

Statistical Analysis Plan:

Sample size: To identify a 2-fold difference, in GMT of IgG between the two intervention groups, with alpha of 0.05 and beta of 0.8, 65 participants in each group are needed. To identify a 3-fold difference before and after the fourth dose for each group, 190 participants are needed. To detect a 20% difference in rate of adverse events between the two intervention arms, we will need 108 participants in each group.

To calculate cumulative incidence subjects will be included in follow up starting from day 8 after receiving the fourth dose. For Control participants, the start day will be on the day the matched intervention arm participant entered the study. Follow up will continue until the end of the study or until becoming positive. Analysis will be performed using two methods: Poisson regression with vaccine groups as the main covariates and calendar day and age-group as confounder covariates; and Cox regression, with calendar days for baseline hazard, left truncation for persons in the vaccine groups who are vaccinated later than January 3rd, including vaccine groups as the main covariates and age-group as confounding covariates. In secondary analyses, the effect of vaccine in two separate periods will be evaluated: from 8-14 days following vaccination and from 15-29 days following vaccination.

Benefits of participating in the study:

The potential benefit of improved protection from SARS-CoV-2. Currently, according the effectiveness of the previous doses, and in the absence of effective drug therapy, it is expected that an additional dose will raise the level of antibodies and thus raise the protection from SARS-CoV-2.

Risks in participating in the study:

The known previous adverse events are expected to occur, these included frequent local reactions (mostly local pain), as well as systemic reactions including fatigue, fever, lymphadenopathy, myalgia, these have typically lasted up to 48 hours. Rarely, myocarditis has occurred (1:6,000-24,000, mostly in young men). It is yet unknown if a 4th dose will have additional adverse reactions.

Some concerns may be raised regarding potential affinity maturation of B cells specific for ancestral variant epitopes encoded by the vaccine, skewing of the T cell response and depletion of memory T cells against other pathogens. The latter point with respect to T cells is not supported by convincing evidence in humans to suggest that this may be the case in the long term nor that it may be detrimental to human health. Many different vaccines are administered at regular intervals in humans and significant detrimental effects to the immune system's response to specific and other pathogens have not been clearly documented.

General comorbidity computer based questionnaire: see Appendix A.

Adverse event questionnaire: See Appendix B.

Appendix A-1 – superiority of 3rd dose (submitted for publication)

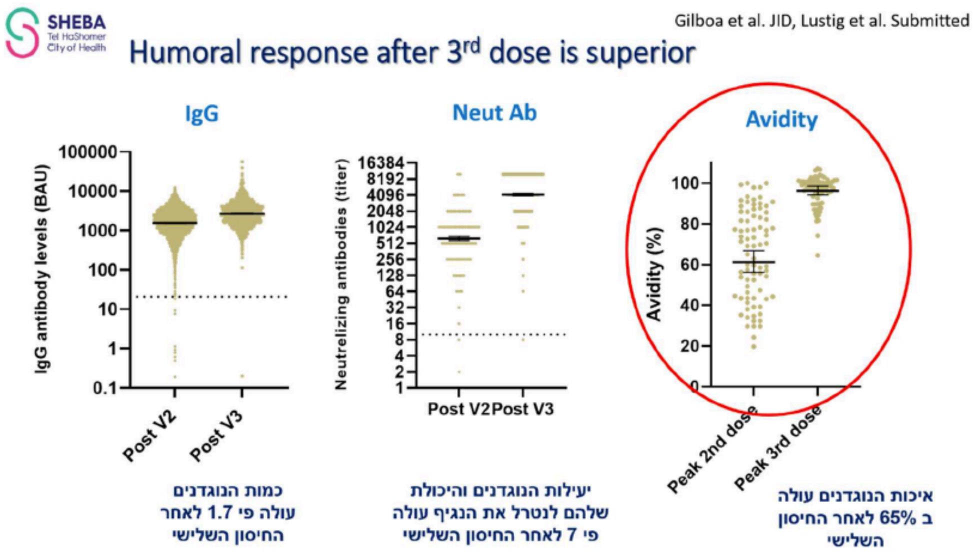

Appendix A2 – Waning immune response after the third dose (preliminary unpublished data)

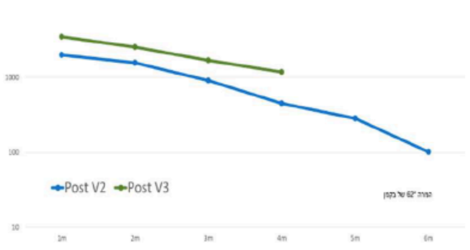

IgG

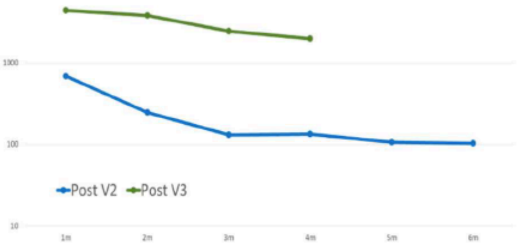

Neutralizing Ab

Appendix A: Study general comorbidity computer-based questionnaire

	Question	Answer1	Answer2
1	What is your date of birth?		
2	What is your gender?	Male	Female
3	What is your current height in m?		
4	What is your current weight in kg?		
5	Did you undergo a SARS-CoV-2 Anti-spike IgG test before receiving the first dose of the vaccine?	Yes	No
6	Do you suffer from systemic hypertension (systolic blood pressure above 140) for which you are pharmaceutically treated?	Yes	No
7	Do you suffer from dyslipidemia (total cholesterol above 200 or LDL cholesterol above 160) for which you are pharmaceutically treated?	Yes	No
9	Do you suffer from an autoimmune disease for which you are pharmaceutically treated?	Yes	No
10	Do you have diabetes (HbA1C>6.5 or fasting blood sugar>126) for which you are pharmaceutically treated?	Yes	No
11	Do you suffer from heart disease for which you are pharmaceutically treated?	Yes	No
12	Do you suffer from lung diseases such as asthma, COPD, and pulmonary fibrosis treated for which you are pharmaceutically treated?	Yes	No
13	Do you suffer from any coagulation disorder resulting in hemorrhage or thrombosis for which you are pharmaceutically treated?	Yes	No
14	Are you immunosuppressed (organ transplant recipient, currently undergoing biologic therapy/chemotherapy, treated with corticosteroids, underwent a splenectomy, or diagnosed with HIV)?	Yes	No
15	Have you ever had a serious allergic reaction (anaphylaxis) that required immediate treatment?	Yes	No
16	Do you have liver disease as cirrhosis, hepatitis, liver cancer, or a metabolic disorder?	Yes	No
17	Do you have kidney disease (a creatinine level of >1.2 mg/dL or GFR<60) for which you are pharmaceutically treated?	Yes	No
18	Are you currently pregnant (as confirmed by a beta-HCG blood test and fetal heartbeat detection on ultrasonography)?	Yes	No

The questionnaire was reviewed and approved by the Institutional review board of the Sheba Medical Center.

Appendix B: Adverse Event computer-based questionnaire

	Question	Answer 1	Answer 2	Answer 3
	**Local symptoms:**			
1	Did you experience pain in the injection site?	Yes	No	
2	Did you experience redness of the injection site?	Yes	No	
3	Did you experience swelling of the injection site?	Yes	No	
4	Did you experience itching at the injection site?	Yes	No	
5	Did you experience local lymph node swelling?	Yes	No	
6	Did you experience other local symptoms? If so, please elaborate	Yes	No	
7	How long did the local symptoms last (in days)?			
8	How would you rate the severity of the local symptoms on a scale of 1-10 (1- mild, 10- severe with major functional impairment)?			
	**Systemic symptoms:**			
9	Did you experience a fever above 37.5? How many days did it last?	Yes	No	Number of days:___
10	Did you experience fatigue? How many days did it last?	Yes	No	Number of days:___
11	Did you experience myalgia? How many days did it last?	Yes	No	Number of days:___
12	Did you experience generalized lymphadenopathy? How many days did it last?	Yes	No	Number of days:___
13	Did you experience headache? How many days did it last?	Yes	No	Number of days:___
14	Did you experience facial nerve palsy? How many days did it last?	Yes	No	Number of days:___
15	Did you experience paresthesia? How many days did it last?	Yes	No	Number of days:___
16	Did you experience an allergic reaction? If so, please elaborate. How many days did those symptoms last?	Yes	No	Number of days:___
17	How would you rate the severity of the systemic symptoms on a scale from 1-10 (1- mild, 10- severe with major functional impairment)?			
18	Did you have any laboratory abnormality? If so, please elaborate.	Yes	No	
19	Did you experience any other symptoms? How many days did they last? If so, please elaborate.	Yes	No	Number of days:___
20	Did you require medical leave due to your symptoms? If so, how long were you absent from work for?	Yes	No	Number of days:___
21	Did you seek medical attention due to your symptoms? If so, please elaborate	Yes	No	
22	Did you require hospitalization?	Yes	No	

The questionnaire was reviewed and approved by the Institutional review board of the Sheba Medical Center

References

1) Amit, S., Regev-Yochay, G., Afek, A., Kreiss, Y. & Leshem, E. Early rate reductions of SARS-CoV-2 infection and COVID-19 in BNT162b2 vaccine recipients. *Lancet* **397**, 875–877 (2021).
2) Modelling the potential consequences of the Omicron SARS-CoV-2 variant in England | CMMID Repository Modelling the potential consequences of the Omicron SARS-CoV-2 variant in England | CMMID Repository. https://cmmid.github.io/topics/covid19/omicron-england.html.
3) Bar-On, Y. M. *et al.* Protection of BNT162b2 Vaccine Booster against Covid-19 in Israel. *N. Engl. J. Med.* **385**, 1393–1400 (2021).
4) Bergwerk, M. *et al.* Covid-19 Breakthrough Infections in Vaccinated Health Care Workers. *N. Engl. J. Med.* **385**, 1474–1484 (2021).
5) Dagan, N. *et al.* BNT162b2 mRNA Covid-19 Vaccine in a Nationwide Mass Vaccination Setting. *N. Engl. J. Med.* **384**, 1412–1423 (2021).
6) Gilboa, M. *et al.* Early Immunogenicity and Safety of the Third Dose of BNT162b2 Messenger RNA Coronavirus Disease 2019 Vaccine Among Adults Older Than 60 Years: Real-World Experience. *J. Infect. Dis.* **225**, 785–792 (2022).
7) Haas, E. J. *et al.* Impact and effectiveness of mRNA BNT162b2 vaccine against SARS-CoV-2 infections and COVID-19 cases, hospitalisations, and deaths following a nationwide vaccination campaign in Israel: an observational study using national surveillance data. *Lancet* **397**, 1819–1829 (2021).
8) Gilbert, P. B. *et al.* Immune correlates analysis of the mRNA-1273 COVID-19 vaccine efficacy clinical trial. *Science* eab3435 (2021) doi:10.1126/science.abm3425.
9) Khoury, D. S. *et al.* Analysis: A meta-analysis of Early Results to predict Vaccine efficacy against Omicron. *medRxiv* (2021) doi:10.1101/2021.12.13.21267748.
10) Levin, E. G. *et al.* Waning Immune Humoral Response to BNT162b2 Covid-19 Vaccine over 6 Months. *N. Engl. J. Med.* **385**, e84 (2021).
11) Nemet, I. *et al.* Third BNT162b2 vaccination neutralization of SARS-CoV-2 Omicron infection. *medRxiv* (2021) doi:10.1101/2021.12.13.21267670.

Statistical Analysis Plan (SAP)

Immunogenicity of a Fourth mRNA COVID-19 vaccine dose;

Homologous (4 doses of BNT162b2) (IRB-8980-21) or heterologous (mRNA1273 following 3 doses of BNT162b2) (IRB-9035-21)

1. Introduction
    a. Major Aims
    b. General study design
    c. Randomization
    d. Blinding

2. Study Objectives and Endpoints
3. Primary Endpoints
4. Secondary Endpoints
5. Exploratory Endpoints
6. Baseline variables
7. Sample Size and Power
8. Safety Monitoring Committee reports
9. Immunogenicity Review
10. General analyses

Introduction

This statistical analysis plan (SAP) provides detailed methodology for summary and statistical analysis of the data collected in studies 8980-21 and 9035-21.

Study Aims: The major objective of this study is to describe the immune responses following a 4th dose of either BNT162b2 or mRNA1273, following 3 doses of BNT162b2, when the third dose was given at least 4 months earlier.

Immune responses to be assessed:

1. Anti-SARS-CoV-2 RBD IgG
2. Anti-SARS-CoV-2 IgA
3. Avidity of IgG antibodies
4. Sars-CoV-2 pseudovirus neutralization
5. SARS-CoV-2 micro-neutralization of different VOCs available and wild type
6. T-cell activation by elispot
7. T-cell activation by flow cytometry
8. B cell repertoire

Of the above, (1), (4) and (5) are primary outcomes and other immune responses are secondary and exploratory outcomes.

The second major objective is to assess safety of a 4th dose. The following solicited local and systemic adverse events will be reported and the proportion of participants reporting each event among the 4th dose recipients will be calculated with its 95%CL.

1. Local reactions, including pain, redness, swelling or itching in the injection site.
2. Systemic reaction, including lymph node swelling (local or generalized), fever > 37.5, fatigue, myalgia, headache, facial nerve palsy, paresthesia, allergic reaction.

Unsolicited AE will also be collected on each visit until visit 5.

Secondary aim: to assess incidence and characteristics of breakthrough infections in 4th dose recipients and compare them to infections in the control group, and between recipients of either vaccine.

Study Design: This is an open-label, non-randomized trial, to assess the immune response of a 4th dose of either BNT162b2 or mRNA1273. We plan to enroll 150-200 participants to each arm. A control group, which will not recieive a 4th dose, but has similar baseline characteristics (from the same eligibile sub-cohort) will be selected for comparisons. For each vaccine recipient 2 matched controls will be selected. Several measures of immunogenicity will be measured and reported:

1. Immune responses before and after the fourth dose will be compared.
2. Immune responses at peak, following 4th dose will be compared to that after 3rd and 2nd doses.
3. Immune responses at peak following the 4th dose, will be compared to that of the control group, which did not receive a 4th dose.
4. Immune responses at peak and the rate of waning of the two vaccines will be compared.

Randomization: This a non-randomized, open-label trial. Designation to an intervention arm will be time dependent. Enrollment to the first intervention arm (BNT162b2) will take place during two consecutive days. Once approval of protocol 9035-21 will be given, two days of enrollment to the second arm will take place.

Blinding: This study is open-labeled, unblended.

Control group: From the sub cohort of all eligible individuals , an age matched (+/-5 years age difference allowed), will be selected in a 1:2 ratio to each participant in each of the intervention arms. A single control will be allowed to serve as a matched control of both intervention groups. Perfect age and gender will be preferred. Control group will be encouraged through text messages, e-mails and telephone calls to obtain a once weekly RT-PCR test for COVID-19. They will be instructed to report any positive COVID-19 test obtained regardless of whether it was taken as part of the study. Additionally they will receive a computer based questionnaire to assess their compliance with COVID-19 testing.

Study Objectives and Endpoints:

Objective	Estimate	Endpoint (outcome measure)
Primary		
To assess immune response to a 4th COVID-19 mRNA vaccine	Geometric mean titer (GMT) at each time point	SARS-CoV-2 RBD-IgG<br>Pseudoneutralization, microneutralizaion of Delta, Omicron and WT strains
	Geometric mean fold rise	Before and after 4th dose
	Geometric mean ratios	Peak of 2nd vs. 3rd vs. 4th dose<br>Intervention group vs. control group<br>Comparison of the 2 intervention groups
To assess safety of a 4th COVID-19 mRNA vaccine	Number of reported AE and the proportion of reported events in each group	Immediate reactions (allergic, within 30 min of vaccine administration)
	Number of reported AE and the proportion of reported events in each group	Solicited local reactions from day 5 to day 21<br>(pain, rash, redness, itching, swelling)

	Number of reported AE and the proportion of reported events in each group	Solicited systemic AE (lymph node swelling, fever > 37.5, fatigue, myalgia, headache, facial nerve palsy, paresthesia, late allergic reaction
Secondary		
To assess immune response to a 4th COVID-19 mRNA vaccine	Geometric mean titer (GMT) at each time point	IgA, T cell activity, avidity, microneutralization of various VOC
To assess SARS-CoV-2 breakthrough infection incidence and characterize breakthrough infections	Breakthrough infection rate in each group (intervention and control)	PCR positivity<br>Rapid Ag test positivity<br>N-gene Ct value<br>COVID-19 symptoms<br>ER or hospitalization

Endpoints:

Primary Endpoints:

Primary Immunogenicity Endpoints:

1) Anti SARS-CoV-2 RBD IgG titers in BAU at each time point. GMT of the groups will be calculated with their 95% confidence interval.
2) Pseudoneutralization titers at each time point for each group will similarly be calculated.
3) Microneutralization titers of Delta, Omicron and WT strains before 4th dose and on each following visit.

Primary Safety Endpoints are as follows:

1) Immediate Reactions – participants will be followed for 30 min after recipient of the vaccine dose. Any immediate reaction will be reported. Primary endpoints that will be reported: The number of immediate reactions in each group, and proportion of immediate reactions. Comparisons between the groups and between age groups will be performed.
2) Local reactions (Pain, redness, swelling, rash or itching) will be collected via an electronic questionnaire from day 5 to day 21. The following measures will be assessed:
    a. Presence or absence
    b. Duration (in days)
    c. Severity (from mild-1, to severe-10)
3) Systemic AE (Lymph node enlargement, Fever>37.5, fatigue, myalgia, headache, facial nerve palsy, paresthesia, late allergic reaction) will be similarly collected via the electronic questionnaire from day 5 to 21. The following measures will be assessed:
    a. Presence or absence
    b. Duration(in days)

    c. Severity (from mild-1, to severe-10), severity will be divided to three categories- 1-3- mild, 4-6 moderate, 7-10 severe

4) Serious AE will be collected through the 180 days of the study. Will be categorized according the MedDRA terms. The safety endpoints "SAEs from the vaccination (received in this study) through 6 months after the vaccination" will be summarized by system organ class and preferred term. Additionally, SAEs will be listed.

Derived variables for presence of each and any local or systemic adverse reaction within 21 days of each vaccination

Variable	Yes (1)	No (0)
Presence of each local reaction on any questionnaire	Participant reports the reaction as "yes" on any of the questionnaires (within 21 days of vaccination)	Participant reports the reaction as "no" on all of the questionnaires (within 21 days of vaccination)
Presence of any local reaction on any questionnaire	Participant reports any local reaction as "yes" on any of the questionnaires (within 21 days of vaccination)	Participant reports all local reactions as "no" on all of the questionnaires (within 21 days of vaccination)
Presence of each systemic AE on any questionnaire	Participant reports the reaction as "yes" on any of the questionnaires (within 21 days of vaccination)	Participant reports the reaction as "no" on all of the questionnaires (within 21 days of vaccination)
Presence of any systemic AE on any questionnaire	Participant reports any systemic AE as "yes" on any of the questionnaires (within 21 days of vaccination)	Participant reports all systemic AE as "no" on all of the questionnaires (within 21 days of vaccination)

Secondary endpoints:

Secondary Immunogenicity Endpoints:

1) Anti SARS-CoV-2 IgA at each time point. GMT of the groups will be calculated with their 95% confidence interval.
2) T cell activity, measured as Tcell activity/10^6 cells. Will be assessed on visits 1, 3, 5 and 7.

Secondary SARS-CoV-2 infection Endpoints

3) SARS-CoV-2 PCR will be performed by nasopharyngeal-oropharyngeal swabs in each visit, and while the pandemic surge is high (>10k newly detected cases/d) active weekly surveillance will continue, for both intervention and control arms.
4) Symptomatic COVID – any SARS-CoV-2 symptom will be requested to be reported. Upon reporting of such a symptom, rapid Ag test and PCR will be performed.

Exploratory endpoints:

Bcell repertoire – initially, on visits 1, 3, 5 and 7, whole blood will be drawn PBMCs separated, and frozen for later evaluation of the B cell repertoire

Baseline variables:

Measurements or samples collected prior to the study vaccination in this study period are considered the baseline data for the assessments.

Demographics, Medical and vaccination History – have been collected upon initial enrollment to the HCW serology study. Yet, the general comorbidity computer-based questionnaire will be filled upon enrollment to this study once again, to reassure full data availablility. These data include date of birth, sex (male or female) , height and weight, comorbidities including hypertention, dyslipidemia, autoimmune disease, diabetes, heart disease, lung disease, coagulation disorder, immunosuppression (including organ transplant recipient, currently undergoing biologic therapy/chemotherapy, treated with corticosteroids, underwent a splenectomy, or diagnosed with HIV), liver disease and kidney disease.

Physical exam by physician / nurse, on vaccination day, including BP, pulse, will be measured following vaccine administration, and if any immediate reaction will develop.

Sample Size and Power

To identify a 2-fold difference, in GMT of IgG between the two intervention groups, with alpha of 0.05 and beta of 0.8, 65 participants in each group are needed. To identify a 3-fold difference before and after the fourth dose for each group, 190 participants are needed. To detect a 20% difference in rate of adverse events between the two intervention arms, we will need 108 participants in each group.

Safety Monitoring Committee reports:

Following each visit a Safety monitoring report will be sent to the committee for evaluation.

Immunogenicity Review

Due to the urgency to inform public health decisions. Following each timepoint, within 2 weeks, as the primary outcomes will be analysed and reported as needed. Data will be disseminated to public health officials as needed and presented to inform the scientific community.

General Analyses

The following descriptive statistics will be used to summarize continuous variables: number of non missing values, mean, standard deviation, median, range.

For binary variables: descriptive statistics for categorical variables (proportions) will be presented in percentage, and the 95%CI, when applicable.

For antibody titers the geometric means will be calculated as the mean of the assay results after making the logarithmic transformation and then exponentiating the mean to express results on the original scale. Two sided 95% CI will be obtained by taking log transformation of assay results, calculating the 95% CI with reference to students t-distribution and then exponentiating the confidence limits.

Geometric mean fold rises –(GMFR) are defined as ratios of the results after vaccination to the results before vaccination. GMFR are limited to participants with nonmissing values at both time points. They are calculated as the mean of the difference of logarithmically transformed assay result and exponentiating the mean. The associated 2-sided 95% Cis will be obtained by constructing Cis using students t-distribution and then exponentiating the confidence limits.

Geometric mean ratios – will be calculated as the mean of the difference of logarithmically transformed assay results and exponentiating the mean. Two-sided Cis will be obtained.

Methods to manage missing data

Participants will receive 4 adverse events electronic questionnaires, participants who will not reply to any of the questionnaires will be contacted by telephone to answer a phone questionnaire, participants who will not reply to any of the questionnaires will be considered as missing data.

Missing serology results will not be imputed.

No additional imputation will be applied to other missing data.

Graphical summaries of the data will be presented using graphpad, including bar plots, scatter plots or line plots.
